# Structural Insights into Layered Tetrahalocuprates(II) Based on Small Unsaturated and Cyclic Primary Ammonium Cations

**DOI:** 10.3390/ma16062236

**Published:** 2023-03-10

**Authors:** Edi Topić, Mirta Rubčić

**Affiliations:** Department of Chemistry, Faculty of Science, University of Zagreb, Horvatovac 102a, 10000 Zagreb, Croatia

**Keywords:** layered structures, crystal structure analysis, powder diffraction

## Abstract

Layered hybrid halometallates represent a promising class of multifunctional materials, yet with many open challenges regarding the interaction between building blocks. In this work, we present a synthetic and analytical methodology for the efficient synthesis and structural analysis of a series of novel tetrahalocuprate(II) hybrids based on small alkylammonium cations. Observed robustness in geometrical motifs provided a platform for crystal structure determination, even from the complex laboratory powder diffraction data. The slight differences in inorganic layer geometry and severe differences in organic bilayer packing are quantified using well-established descriptors for these materials, and dependences of geometric parameters on anion and cation choice are accounted for. Temperature dependence of structural parameters for one of the tetrachlorocuprate hybrids that was chosen as a model unveils a possible geometrical origin of thermochromism in these materials.

## 1. Introduction

Over the recent years, layered hybrid organic-inorganic halometallates (HOIHs) have emerged as ubiquitous materials offering a wide range of promising applications [1,2,3,4,5]. In comparison with their geometrically restricted 3D relatives, layered (or often termed as 2D) HOIHs show greater structural flexibility and stability, while delivering the opportunity to incorporate larger organic cations in the structures. As a result, by the proper selection of the building blocks, inorganic cations, halide anions, and inorganic cations, diverse structures can be achieved, thus offering a platform for the development of materials with targeted functionalities [6,7,8,9,10,11,12].

According to the plane of the parent 3D perovskite structure along which they have been cut from, 2D HOIHs, with the general formula (A^n+^)_2/n_BX_4_ (A^n+^ = organic cation (usually amine), B^2+^ = inorganic cation; X^−^ = halogen atom), can be classified into (100), (110) or (111) ones. The first option provides 2D structure with flat inorganic BX_4_^2−^- sheets, unlike the remaining two types where corrugated and deformed layers are observed. Furthermore, in the (100) family, one distinguishes “eclipsed” structures, which include monolayers of dications, and the “staggered” ones, relying on double layers of monocations. In an alternative description, the “eclipsed” (100) types can be considered as Dion-Jacobson structures, while the “staggered” (100) assemblies are a special case of Ruddelsden-Popper phases (in both cases thickness of inorganic layers is one octahedral unit) [13,14,15].

Among 2D HOIHs Cu(II)-based ones have lately shown considerable potential, as it was demonstrated that they can be considered as equally efficient but environmentally more benign options to the Pb-based HOIHs which were typically engaged in photovoltaic applications [16,17]. On the other hand, Cu(II)-based HOIHs can display different magnetic-based phenomena like chiral ferromagnetism or multiferroic behavior [18,19,20,21], along with the stimuli-responsive behavior like thermochromism [22,23,24,25,26,27], which extends the paths of their application. While trying to comprehend the intimate structure–property relationship in HOIHs, one becomes aware of a multitude of factors that are coupled with the particular structural type, and subsequently its properties and potential for application. Unveiling this connection becomes especially challenging when the inorganic, and particularly organic building blocks in the system, are geometrically less constrained than in their purely inorganic counterparts, as is the case for the low-dimensional Cu(II)-based HOIHs [13,28]. Taken together, it would be beneficial to systematically explore the relationship between targeted composition, crystal structure/geometric features, and exploitable properties.

As a contribution to this effort, herein, we present a comprehensive solid-state study focusing in particular on the synthesis and structural aspects of a series of layered tetrahalocuprates(II) (CuCl_4_^2−^, CuBr_4_^2−^) based on small unsaturated amines (allylamine (aa) and aminoacetonitrile (aacn)) and cyclic monoamines (cyclopropylamine (cpa), and cyclopropylmethylamine (cpma)). Namely, by choosing halocuprates(II) as inorganic building blocks, with their pronounced Jahn-Teller distortion, we were able to examine the impact of halide anions and organic cations on the degree of distortion, which is relevant for the development of this class of HOIHs as magnetic materials. On the other hand, small monoamines with minimal functionalities, simultaneously enabling without exception formation of layered structures, gave us the opportunity to explore the impact of subtle structural changes on the geometry of the inorganic layers in this class of 2D HOIHs. Moreover, we demonstrate that the robustness of geometrical parameters of 2D HOIHs can be exploited for posing a plausible structural model which can be refined against PXRD data. Finally, we present temperature-dependent structural changes in a prepared tetrachlorocuprate(II) hybrid, pointing to a possible geometrical origin of observed themochromism in layered CuCl_4_ HOIHs_._

## 2. Materials and Methods

Cyclopropylamine (cpa), cyclopropylmethylamine (cpma), allylamine (aa) hydrochloride, aminoacetonitrile (aacn) hydrochloride, and anhydrous copper(II) bromide were purchased from Fluorochem Ltd. (Hadfield, UK) and used without further purification. Anhydrous copper(II) chloride was prepared by reaction of thionyl chloride (Merck) with copper(II) chloride dihydrate (Kemika, Zagreb, HR, Croatia). Dichloromethane (Kemika, Zagreb, HR, Croatia), aminoacetonitrile (Kemika, Zagreb, HR, Croatia), hexane (Kemika, Zagreb, HR, Croatia), and 4 mol dm^−3^ solution of hydrogen chloride in dioxane (Sigma Aldrich, St. Louis, MO, USA) were used without further purification. Hydrochloric acid (18% *w*/*w*) was prepared by diluting concentrated hydrochloric acid (Kemika, Zagreb, HR, Croatia) with distilled water. Anhydrous copper(II) chloride [29] and triphenylphosphinium bromide [30] (PPh_3_·HBr) were prepared according to literature procedures. Elemental analyses (C, H, N, Cu) were provided by the Analytical Services Laboratory of the Ruđer Bošković Institute, Zagreb, Croatia.

### 2.1. Synthesis of Alkylammonium Halogenides

Due to exceptional hygroscopicity of ammonium halogenides, the following procedure was used to prepare the salts in anhydrous conditions.

Alkylammonium chlorides from free base: 10 mmol of alkylamine free base was dissolved in 5 mL of cold dichloromethane. Then, 3 mL of 4 mol dm^−3^ solution of hydrogen chloride in dioxane was added dropwise to the amine solution with violent stirring. The chloride salt precipitates instantly upon addition, and was vacuum filtered over sintered glass filter, washed with 2–3 mL of dichloromethane and dried under stream of argon gas. Once isolated, the salt was kept in desiccator over anhydrous calcium chloride. The salts prepared by this method were cpa·HCl (elem. analysis [C_3_H_8_NCl] calcd. C 38.51%, H 8.64%, N 14.97%; found C 38.00%, H 8.58%, N 14.92%) and cpma·HCl (elem. analysis [C_4_H_10_NCl] calcd. C 44.65%, H 9.39%, N 13.02%; found C 44.21%, H 9.71%, N 12.85%).

Alkylammonium bromides from free base: 10 mmol of alkylamine free base was dissolved in 5 mL of cold dichloromethane. Then, solution of triphenylphosphinium bromide in cold hexane (15 mmol in 20 mL) was added at once to the amine solution, and the solution was thoroughly mixed. The bromide salt precipitated within a few minutes, and was vacuum filtered over sintered glass filter, washed with 2 × 10 mL of hexane and dried under the stream of argon. Once isolated, the salt was kept in desiccator over anhydrous magnesium sulfate. The salts prepared by this method were cpa·HBr (elem. analysis [C_3_H_8_NBr] calcd. C 26.10%, H 5.85%, N 10.15%; found C 25.63%, H 6.52%, N 9.86%) and cpma·HBr (elem. analysis [C_4_H_10_NBr] calcd. C 31.59%, H 6.64%, N 9.21%; found C 30.75%, H 6.29%, N 8.82%).

Alkylamine hydrobromide from alkylamine hydrochloride: 10 mmol of alkylamine hydrochloride was suspended in 10 mL of dichloromethane, and 1 mL of methanol was added to the solution. Then, 1.38 g (10 mmol) of potassium carbonate was added to the suspension, and suspension was stirred for 10 min. The suspension was filtered through sintered glass filter, and to the filtrate a solution of triphenylphosphinium bromide in cold hexane (15 mmol in 20 mL) was added at once. Alkylamine hydrobromide precipitated within a few minutes, and was vacuum filtered over sintered glass filter, washed with 2 × 10 mL of hexane and dried over stream of argon gas. Once isolated, the salt was kept in desiccator over anhydrous magnesium sulfate. The salts prepared by this method were aa·HBr (elem. analysis [C_3_H_8_NBr] calcd. C 26.10%, H 5.85%, N 10.15%; found C 25.61%, H 5.57%, N 10.46%) and aacn·HBr (elem. analysis [C_2_H_5_N_2_Br] calcd. C 17.53%, H 3.96%, N 20.45%; found C 17.14%, H 4.45%, N 19.92%).

### 2.2. Crystallization of Tetrachlorocuprate(II) Salts from Solution

Briefly, 1 mmol of alkylammonium chloride and 0.5 mmol of copper chloride dihydrate were dissolved in the minimal amount of hydrochloric acid (18% *w*/*w*). Then, the solutions were combined in a glass beaker, which was closed with a stopper containing small holes to allow evaporation. In the case of aacn_2_CuCl_4_ and cpa_2_CuCl_4_ the experiments resulted in crystals of decent quality, while the experiments with cpma_2_CuCl_4_ yielded highly mosaic crystals. In the case of aa_2_CuCl_4_ salt, powdered material was obtained. Similar experiments were attempted in order to obtain crystals of tetrabromocuprate salts. However, such procedures have not yielded solid products, possibly due to exceptional solubility of these salts.

### 2.3. Mechanochemical Synthesis

Briefly, 1 mmol of alkylammonium halogenide and 0.5 mmol of anhydrous copper halogenide were quickly weighted in a PTFE milling jar. Before closing, a 10 mm alumina milling ball and 10 µL of acetonitrile was added in the jar. The milling was performed on Retsch MM 200 vibrational mill, at 25 Hz for 10 min. After the milling, the product was transferred in a glass bottle and kept in desiccator over calcium chloride (for chloride salts) and magnesium sulfate (for bromide salts). The compounds prepared by this method were tetrachlorocuprates aa_2_CuCl_4_ ([C_6_H_16_N_2_Cl_4_Cu] calcd. Cu 19.8%, found 19.5%), aacn_2_CuCl_4_ ([C_4_H_10_N_4_Cl_4_Cu] calcd. Cu 19.9%, found 19.7%), cpa_2_CuCl_4_ ([C_6_H_16_N_2_Cl_4_Cu] calcd. Cu 19.8%, found 18.9%), cpma_2_CuCl_4_ ([C_8_H_20_N_2_Cl_4_Cu] calcd. Cu 18.2%, found 18.0%), and tetrabromocuprates aa_2_CuBr_4_ ([C_6_H_16_N_2_Br_4_Cu] calcd. Cu 12.7%, found 11.1%), aacn_2_CuBr_4_ ([C_4_H_10_N_4_Br_4_Cu] calcd. Cu 12.8%, found 12.7%), cpa_2_CuBr_4_ ([C_6_H_16_N_2_Br_4_Cu] calcd. Cu 12.7%, found 11.5%), cpma_2_CuBr_4_ ([C_8_H_20_N_2_Br_4_Cu] calcd. Cu 12.0%, found 10.8%).

### 2.4. FTIR Spectroscopy

The FTIR spectra were collected in attenuated total reflectance (ATR) mode on Nicolet iS50 spectrometer (Thermo Scientific, Waltham, MA, USA). Mid-IR data was collected in 4000–400 cm^−1^ range by averaging 8 scans, while far-IR data was collected in 600–120 cm^−1^ by averaging 32 scans. FTIR data for alkylammonium halogenides and alkylammonium tetrahalocuprates are included in Supplementary Materials, Figures S1–S12.

### 2.5. Single-Crystal X-ray Diffraction (SCXRD)

The diffracted intensities were collected via *ω*-scans on an Rigaku Synergy diffractometer equipped with 4-circle kappa geometry goniometer, HyPIX-6000 detector and microfocus Cu K_α_ source (*λ* = 1.54184 Å) at 298(2) K. The collected data was reduced using the CrysAlisPro v171.42.49 [31] software package. The initial structural model was found using SHELXT v2019 [32] with dual space methods. The refinement procedure by full-matrix least-squares methods based on *F*^2^ values against all reflections included anisotropic displacement parameters for all non-H atoms. Hydrogen atoms attached to carbons were placed in geometrically idealized positions and were refined using the riding model with *U*_iso_ = 1.2*U*_eq_ of the connected carbon atom or as ideal CH_3_ groups with *U*_iso_ = 1.5*U*_eq_. Hydrogen atoms attached to heteroatoms were located in the difference Fourier maps at the final stages of the refinement procedure and refined with *U*_iso_ = 1.2*U*_eq_. All refinements were performed using SHELXL v2019 [33]. The SHELX programs operated within the Olex2 v1.5 suite. Geometrical calculations were performed using the PLATON v201118, and graphics were prepared using VESTA v3.5.7 [34] and Mercury v2022.3.0 [35] software. Detailed experimental and crystallographic data can be found in Supplementary Materials, under Crystallographic and structural data.

### 2.6. Powder X-ray Diffraction

Powder diffraction data were collected on Panalytical Empyrean diffractometer equipped with Mo Kα source with elliptical W/Si focusing mirror and GaliPIX3D HPC detector in capillary transmission mode, using 0.5 mm thin-walled borosilicate glass capillaries. Indexing and refinement was done in TOPAS Academic software v.5 [36]. Background scattering intensities were modelled as linear combination of Chebyshev polynomials. Instrument-related and sample-related peak convolution parameters were refined using fundamental parameters approach [37]. Organic cation geometry was fully constrained on bond lengths and angles, but torsion angles were refined freely, where bond rotation was chemically meaningful. Inorganic anion geometry was constrained considering the point group of metal atom Wyckoff position. Orientation and position of fragments, as well as the values of unconstrained parameters were found using a simulated annealing procedure as implemented in TOPAS. Final refinement was conducted by simultaneously refining all instrumental, sample, and structure parameters. More information regarding collected data, refinement, and TOPAS input files can be found in the Supplementary Materials, under Rietveld refinement results and TOPAS input files. Graphical fit results can be found in Supplementary Materials, Figures S13–S20. Powder diffraction data for alkylammonium halogenides can be found in Supplementary Materials, Figure S21.

### 2.7. Thermal Analysis

The obtained compounds were analyzed on Perkin Elmer DSC8500 differential scanning calorimeter (Waltham, MA, USA). Heat flow was measured from −70 °C to 120 °C (upper limit set by anticipated sample decomposition temperature), with heating rate of 10 °C/min under dynamic nitrogen atmosphere with flow of 20 mL/min. The sample was prepared by weighing a small amount (2–5 mg) of compound in an aluminum pan, which was then closed with aluminum lid and crimped. Measured thermograms are presented in Supplementary Materials, Figures S22 and S23.

## 3. Results and Discussion

**Scheme 1:**
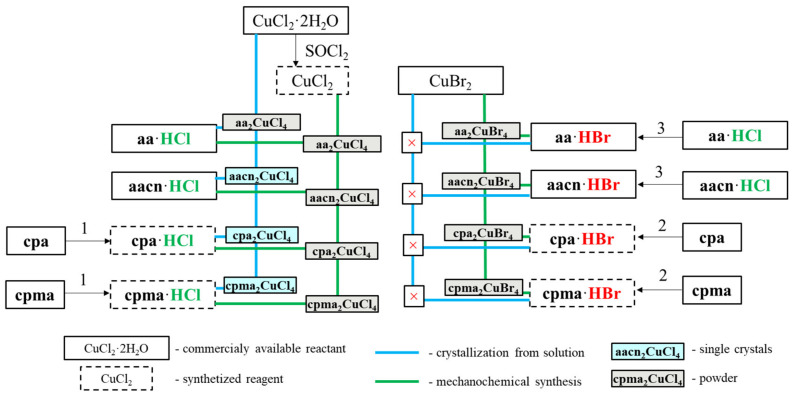
Results of synthetic procedures performed in this work. Alkylammonium salts were successfully prepared by (1) reaction of free base with HCl/dioxane, (2) reaction of free base with PPh_3_·HBr and (3) conversion of hydrochloride to free base and subsequent reaction with PPh_3_·HBr. Crystallization from solution yielded single crystals for three compounds and powder for one compound, but only for tetrachlorocuprates. Mechanochemical synthesis yielded the targeted products in all instances.

Even though one could expect a similar chemical behavior for the series of HOIHs presented herein, a remarkable difference in the solubilities between tetrachlorocuprates(II) and tetrabromocuprates(II) was established. While the solution based synthesis yielded the corresponding tetrachlorocuprates(II), tetrabromocuprates(II) demonstrated exceptional solubility in aqueous solutions, which hindered their isolation from such media. This stimulated us to explore potential of mechanochemical synthesis, for which a simple procedure for the preparation of alkylammonium halogenides was developed. Reacting anhydrous copper halogenides and alkylammonium halogenides by milling proved to be a fruitful alternative towards tetrabromocuprates(II), albeit at the expense of obtaining only powdered products (Scheme 1). In the case of tetrachlorocuprates(II), identical products were obtained from mechanochemical synthesis as from the solution-based one (Scheme 1).

Such a scenario posed certain limitations for the structural analysis in the case of tetrabromocuprates(II), as the structural features could only be obtained from the powder diffraction data, which intrinsically give less information in comparison to SCXRD. This becomes even more pronounced when elucidating structures from laboratory X-ray diffraction data, where non-monochromatic source spectrum and instrument convolutions lead to complex peak shapes and the severe overlap of reflections [38].

Nevertheless, based on the reported SCXRD structures of layered tetrahalocuprate salts, one can set useful constraints regarding the unit cell choice, space group symmetry and positions of inorganic and organic fragments in the structure. Namely, the 2D HOIH structure type is defined by organic cation (bi)layers sandwiched between layers of edge-sharing halometallate octahedra. If the material is periodic in three dimensions, the unit cell must be commensurate with intralayer and interlayer dimensions, and the space group setting must be compatible with the stoichiometry and point group symmetry of the building blocks. When considering the reported structures of tetrahalocuprate HOIHs, the most frequent cell choices for layered tetrahalocuprates are monoclinic or orthorhombic cells with metric a×a×nc or $\sqrt{2}a\times \sqrt{2}a\times \mathrm{nc}$, with a being the shortest metal-metal in-layer distance and nc being a whole multiple of shortest metal-metal out-of-layer distance. This is similar to their lead halide counterparts [15]. Thus, during the indexation procedure, unit cells compatible with layered HOIH structure can be selected readily. Moreover, based on the selected cell, one can limit the number of plausible space groups based on the compatibility of Wyckoff positions of metal, halogen, and organic ions with the stoichiometry of the compound, and by considering that the CuX_6_ octahedra is usually (only) centrosymmetric (i.e., point group $\bar{1}$).

**Figure 1:**
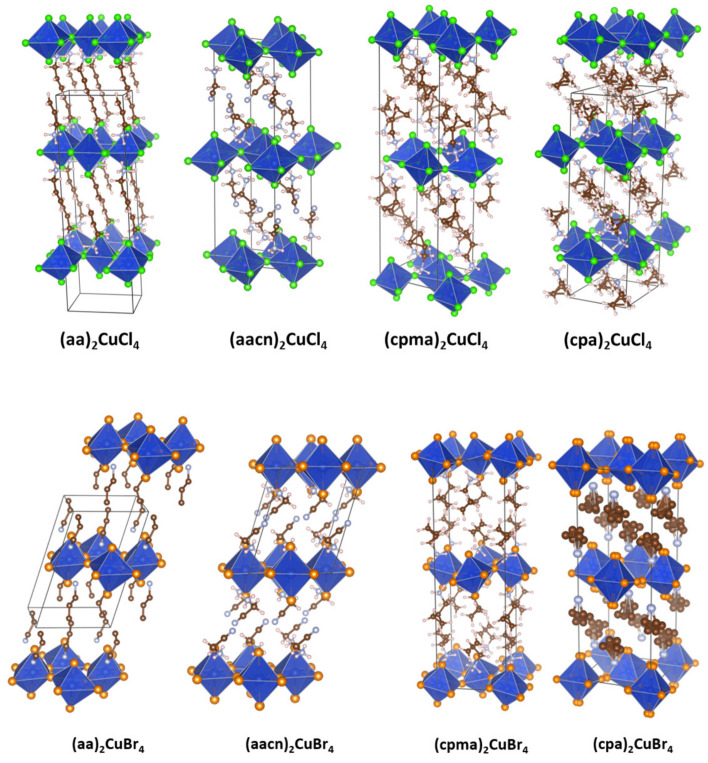
Packing in the crystal structures of (**top**) alkylammonium tetrachlorocuprates(II) and (**bottom**) alkylammonium tetrabromocuprates(II) prepared in this work. Unit cell is highlighted with black lines. Centers of blue octahedra are occupied with copper ions. Chloride and bromide ions are drawn as green and orange spheres, respectively. Carbon, nitrogen and hydrogen atoms are presented as brown, light blue and gray spheres, respectively. The anions pack in layers of corner-sharing octahedra, while the cations form a bilayer between the inorganic layers, akin to hybrid Ruddlesden-Popper phases.

By following these considerations, powder data of aa_2_CuCl_4_ and the four prepared tetrabromocuprate(II) HOIHs were successfully indexed with a HOIH-compatible cell, best-fitting space group was determined, and reasonable structural models were proposed for all five compounds (Figure 1, SI, Tables S4 and S5), despite the limited information available in the collected diffraction data.

The plausibility of chosen structural models is supported and justified, besides the comparison with the SCXRD data in some cases, also by the relevant spectroscopic data (SI, Figures S1–S12). Namely, the analysis of the relevant bands, observed at ≈270 cm^−1^ for tetrachlorocuprates(II) and at 200 cm^−1^ for tetrabromocuprates(II), in the Far-IR spectra confirms the presence of MX_4_ subunits as the main building block of inorganic layers in all compounds [39]. Finally, it should be highlighted that for all compounds investigated herein, cell choices have a metric of $\sqrt{2}a\times \sqrt{2}a\times c$ or $\sqrt{2}a\times \sqrt{2}a\times 2c$ (Figure 1, SI, Tables S1–S5).

**Figure 2:**
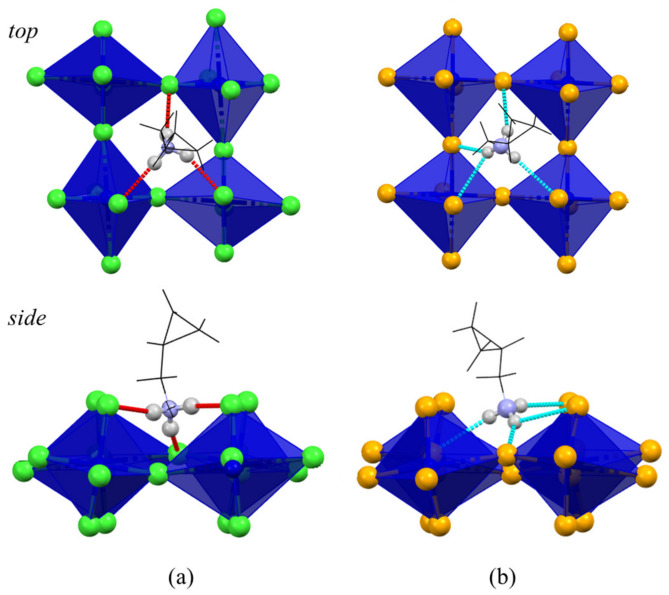
Top and side views of hydrogen bond anchoring of organic cation in (**a**) cpma_2_CuCl_4_ and (**b**) cpma_2_CuBr_4_.

Having such set of compounds at our hands motivated us to explore and compare their underlying structures in more detail. The robustness of the main assembly is evident as all structures contain inorganic layers of edge-sharing, Jahn-Teller distorted octahedra (Table 1, parameter Δ). Furthermore, all structures can be distinguished as Ruddlesden-Popper ones, with the characteristic offset of the neighboring inorganic layers (Table 1, parameter LSF *t*_1_, t_2_ = ≈0.5, ≈0.5;), typical for the HOIHs of the A_2_MX_4_ stoichiometry. When considering the organic part of the structure, one observes a similar scenario in all cases, in that the cations are anchored to the inorganic layers via ammonium-halogenide hydrogen bonds. In other words, ammonium nitrogen atoms reside in close proximity to the centroid of axial halogenide ions (Figure 2 and Table 2, column cation penetration, Supporting Information, Tables S6–S13).

While there is evident robustness and structural similarity in this series of compounds that can be beneficial from a predictive material design viewpoint, one finds significant differences between the observed structures, which can be distinguished into cation-dependent and anion-dependent ones. 

**Figure 3:**
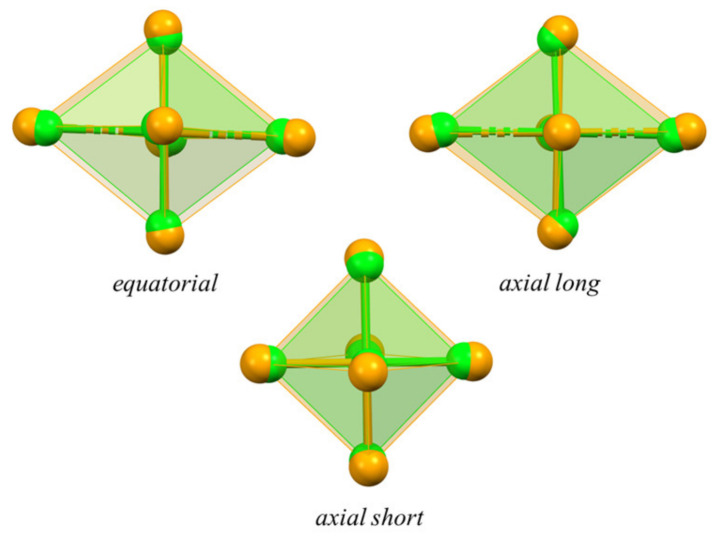
Projections of CuX_6_ octahedra in aacn_2_CuCl_4_ (green) and aacn_2_CuBr_4_ (orange) on equatorial plane and two perpendicular axial planes. Cu-Br bonds are usually significantly longer than appropriate Cu-Cl bonds in these compounds.

Anion-dependent differences arise from the fact that CuCl_6_ and CuBr_6_ moieties have significantly different Cu-X bond lengths, i.e., Cu-Br bond lengths are around 5–10% longer than equivalent Cu-Cl bond lengths, due to slightly larger ionic radius of the bromide ion [40]. Consequentially, the shortest Cu-Cu distances are greater by the same extent in tetrabromocuprates (Figure 3, Supplementary Materials, Tables S6–S13). The Jahn-Teller distortion, on the other hand, is comparable in both chemistries, as it is largely a property of the metal ion. Another difference is the predominance of centrosymmetric octahedra in chlorocuprates, and the appearance of non-centrosymmetric octahedra in aa_2_CuBr_4_ and cpma_2_CuBr_4_, which is possibly due to the (lack of) symmetry imposed by the best-fitting space group choice, and is supported by several reported structures of the similar type [41,42,43,44].

On the other hand, cation-dependent differences are not that obvious. The interlayer distance as a function of cation size is comparable between the chloro- and bromocuprates (Table 2, parameter *d*(Cu-Cu)_oop_). However, at first, it is not clear what cation property defines the observed interlayer distances. Our interpretation is that aacn cation, having a non-bulky nitrile moiety, can pack more closely in the organic bilayer than the rest of the cations, while the packing efficiency of cpma cation can be expected to be the worst, leading to largest interlayer distances and lowest density. In the case of aa based hybrids, an additional torsional degree of freedom and two hydrogen atoms at the terminus of the cation drastically enlarges its bulkiness compared to aacn, so the packing efficiencies and crystal densities are lower. The same could be said for cpa, but in lieu of torsional freedom, this cation experiences rotational disorder, as supported by several cases in reported structures of HOIHs containing cpa cation [45,46,47,48]. 

Unfortunately, there is no obvious metric by which packing efficiency of the cations can be quantified; it seems that it depends upon the complex interplay between weak attractive and repulsive interactions in the organic bilayer and strong directed hydrogen-bond anchoring of the ammonium group, which is also strongly dependent on the inorganic layer geometry. Other easily calculated geometrical parameters, such as cation penetration [13] and angle between the C-NH_3_^+^ bond and the inorganic layer (which quantifies the cation orientation), do not seem to correlate to the packing efficiency, or to any other geometrical parameter for that matter.

Finally, for this type of layered compounds, the dependance of the related structural parameters upon external conditions, such as temperature or pressure, is expected and frequently encountered. The thermochromism, as a stimuli-responsive property, has gained particular attention over the years due to its promising applicability in the field of sensors [49,50]. The potential for thermal sensor applications, as well as thermal management applications, has been demonstrated in Cu-based hybrids showing reversible and irreversible thermochromic behavior [51,52]. Thermochromism in hybrid materials is often coupled with the structural distortions of a certain type and is most frequently evident as continuous thermochromism. However, there are still many challenges in this respect, which are demonstrated by examples of reversible and irreversible thermochromism of the layered tetrachlorocuprates [22,23,24,25,26,27]. Particularly, it is not clear what structural changes lead to such strong and ubiquitous effect in this class of materials. This is aggravated by the fact that most of the investigated compounds show structural phase transitions across the relevant temperature range, preventing the accurate comparison of geometrical data.

**Figure 4:**
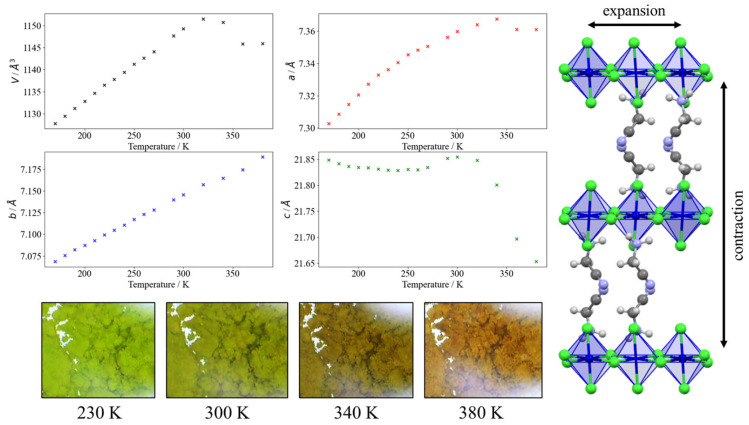
Unit cell parameters of aacn_2_CuCl_4_ as a function of temperature. The parameters follow a linear trend, up to ~320 K, after which cell volume, and axes *a* and *c* decrease. This can be interpreted as the layer distorting itself, and organic cations coming closer together at high temperatures.

**Figure 5:**
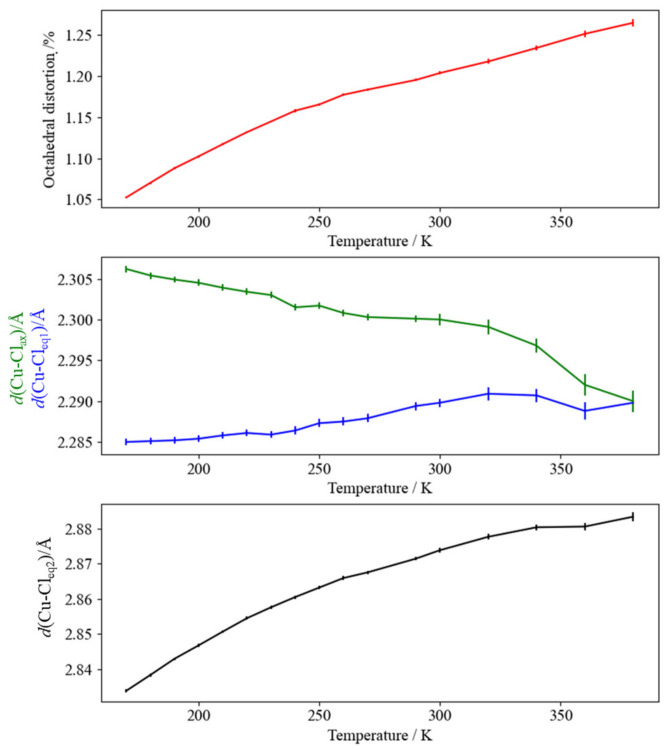
Parameters of octahedral geometry in aacn_2_CuCl_4_ as a function of temperature (±1σ error bars included). Octahedral distortion monotonically increases through the temperature range. The square planar geometry of CuCl_4_ unit becomes more symmetrical at high temperatures, but the elongation perpendicular to the plane becomes more pronounced. The symmetrization of the square planar anion could be the underlying mechanism behind observed thermochromism.

However, for tetrachlorocuprate HOIHs prepared herein, no signatures of abrupt phase transitions were found, as evidenced by uneventful DSC thermograms for majority of them (Supplementary Materials, Figures S22 and S23). Having the high-quality single-crystals available, we conducted temperature-dependent experiments and extracted, for aacn_2_CuCl_4_ as a model system, the relevant structural data (Figure 4 and Figure 5, Supplementary Materials, Tables S2 and S14). The aacn_2_CuCl_4_ was chosen as an ideal candidate for precise determination of temperature-dependent geometric changes in the organic and inorganic moieties, as the structural changes are smooth in the evaluated temperature range, and the crystal structure is non-disordered. It can be seen that unit cell parameters increase linearly from 170 K to approximately 320 K, when, curiously, interlayer distance and unit cell volume begin to decrease. The most obvious structural change is the apparent symmetrization of square-planar CuCl_4_ moiety (Figure 5, SI, Table S14), which could be the underlying cause of thermochromism for this compound. Moreover, the linear increase of the octahedral distortion, i.e., area of octahedral voids in which cation resides, could lead to organic bilayer compression due to more efficient cation packing, which could account for the observed nonlinearity of the unit cell parameters at high temperatures. This is supported by previously reported explanations of thermochromism in layered tetrachlorocuprate(II) HOIHs, and can be related to the similar mechanism of Jahn-Teller suppression in a reported piezochromism study [26]. However, to the best of our knowledge, data presented herein represent the first instance of temperature-dependent structural evolution for A_2_CuCl_4_ layered HOIH modelled after SCXRD data.

To conclude, by evaluating the series of the title HOIHs, based on small unsaturated and cyclic primary ammonium cations, we have unveiled the robust similarities and subtle differences in their crystal structures, and provided a set of guidelines for the synthesis and elucidation of the corresponding structural data. Even though the geometrical features of these compounds are predictable along two in-layer dimensions, the features along the third, cation dependent dimension, show intriguing variability. This certainly provides motivation for further efforts in the theoretical modelling of these compounds, even at a basic geometrical level. Furthermore, we quantified the slight temperature-dependent variations in the crystal structure of a model tetrachlorocuprate(II) HOIH in order to provide a geometrical background for the observed thermochromism. Indeed, more temperature-dependent studies are needed to fully discern the important structural changes in this type of compounds, but this example points out the possible direction for future studies. 

## Figures and Tables

**Table 1:** Geometrical parameters of octahedral CuCl_6_ units in the structures of prepared compounds. $\bar{d}$(Cu-Cu)_ip_ represents in-plane distance between the neighbouring copper ions, $\bar{d}$(Cu-X)_eq_ represent average in-plane (equatorial) copper-halogenide distances and $\bar{d}$(Cu-X)_ax_ represent average out-of-plane (axial) copper-halogenide distances, Δ/% represents octahedral distortion [28], LSF represents layer translation values [13].

	$\bar{d}$(Cu-Cu)_ip_/Å	$\bar{d}$(Cu-X)_eq_/Å	$\bar{d}$(Cu-X)_ax_/Å	Δ/%	**LSF *t*_1_, *t*_2_**
(aa)_2_CuCl_4_	5.27	2.35, 2.93	2.29	1.46	0.43, 0.44
(aacn)_2_CuCl_4_	5.13	2.29, 2.87	2.30	1.21	0.47, 0.47
(cpma)_2_CuCl_4_	5.22	2.28, 2.98	2.31	1.72	0.46, 0.49
(cpa)_2_CuCl_4_	5.25	2.29, 3.01	2.29	1.88	0.4, 0.42
(aa)_2_CuBr_4_	5.43 **	2.46, 2.98 **	2.33	1.37	0.28, 0.48
(aacn)_2_CuBr_4_	5.61	2.49, 3.20	2.43	2.02	0.28, 0.38
(cpma)_2_CuBr_4_	5.57 **	2.46, 3.19 **	2.40	2.10	0.49, 0.50
(cpa)_2_CuBr_4_	5.62	2.48, 3.20	2.44	2.02	0.5, 0.5 *

* estimation, calculation not possible due to disorder; ** the metal ion is not positioned on the inversion center.

**Table 2:** Cation-dependent structural parameters observed in the crystal structures of alkylammonium tetrahalocuprates prepared in this work. *d*(Cu-Cu)_oop_ represents interlayer distance, *cation penetration* represents distance between the ammonium nitrogen atom and a plane spanned by axial halogenide ions [13], ∠(C-N_am_-layer) represents the angle between C-NH_3_^+^ bond and plane spanned by metal ions, and *ρ_cryst_* represents density of the crystal calculated from structural data.

	*d*(Cu-Cu)_oop_/Å	Cation Penetration/Å	∠(C-N_am_-Layer)/°	*ρ_cryst_*/g cm^−3^
(aa)_2_CuCl_4_	*a*/2, 12.05	0.37	52	1.600
(aacn)_2_CuCl_4_	*c*/2, 10.93	0.07	24	1.846
(cpma)_2_CuCl_4_	*a*/2, 13.39	0.20	38	1.597
(cpa)_2_CuCl_4_	*a*/2, 11.21	0.19	21	1.734
(aa)_2_CuBr_4_	*a*, 12.49	0.08, 0.59	48, 28	2.344
(aacn)_2_CuBr_4_	*a*, 10.08	0.08	71	2.735
(cpma)_2_CuBr_4_	*c*/2, 13.00	0.13	28	2.089
(cpa)_2_CuBr_4_	*c*/2, 10.93	0.01	23	2.326

## Data Availability

The data presented in this study are available as a part of the article and in its supplementary material. Additionally, CCDC 2238569-2238587, 2238812-2238816 contain the supplementary crystallographic data for this paper. These data can be obtained free of charge via http://www.ccdc.cam.ac.uk/conts/retrieving.html (3 February 2023) (or from the Cambridge Crystallographic Data Centre, 12, Union Road, Cambridge CB2 1EZ, UK; fax: +44-1223-336033).

## Supplementary Materials

The supporting information can be downloaded at: https://www.mdpi.com/article/10.3390/ma16062236/s1.

## References

[1] Babu R., Giribabu L., Singh S.P. (2018). Recent Advances in Halide-Based Perovskite Crystals and Their Optoelectronic Applications. Cryst. Growth Des..

[2] Privitera A., Righetto M., Cacialli F., Riede M.K. (2021). Perspectives of Organic and Perovskite-Based Spintronics. Adv. Opt. Mater..

[3] Wang G., Mei S., Liao J., Wang W., Tang Y., Zhang Q., Tang Z., Wu B., Xing G. (2021). Advances of Nonlinear Photonics in Low-Dimensional Halide Perovskites. Small.

[4] Wang Y., Song L., Chen Y., Huang W. (2020). Emerging New-Generation Photodetectors Based on Low-Dimensional Halide Perovskites. ACS Photonics.

[5] Stylianakis M., Maksudov T., Panagiotopoulos A., Kakavelakis G., Petridis K. (2019). Inorganic and Hybrid Perovskite Based Laser Devices: A Review. Materials.

[6] Lin H., Zhou C., Tian Y., Siegrist T., Ma B. (2018). Low-Dimensional Organometal Halide Perovskites. ACS Energy Lett..

[7] Zhou C., Lin H., He Q., Xu L., Worku M., Chaaban M., Lee S., Shi X., Du M.-H., Ma B. (2019). Low Dimensional Metal Halide Perovskites and Hybrids. Mater. Sci. Eng. R Rep..

[8] Mao L., Stoumpos C.C., Kanatzidis M.G. (2019). Two-Dimensional Hybrid Halide Perovskites: Principles and Promises. J. Am. Chem. Soc..

[9] Saidaminov M.I., Mohammed O.F., Bakr O.M. (2017). Low-Dimensional-Networked Metal Halide Perovskites: The Next Big Thing. ACS Energy Lett..

[10] Pedesseau L., Sapori D., Traore B., Robles R., Fang H.-H., Loi M.A., Tsai H., Nie W., Blancon J.-C., Neukirch A. (2016). Advances and Promises of Layered Halide Hybrid Perovskite Semiconductors. ACS Nano.

[11] Jin C., Li F., Yang Z., Pan S., Mutailipu M. (2022). [C_3_N_6_H_7_]_2_ [B_3_O_3_F_4_(OH)]: A New Hybrid Birefringent Crystal with Strong Optical Anisotropy Induced by Mixed Functional Units. J. Mater. Chem. C Mater..

[12] Jin C., Zeng H., Zhang F., Qiu H., Yang Z., Mutailipu M., Pan S. (2022). Guanidinium Fluorooxoborates as Efficient Metal-Free Short-Wavelength Nonlinear Optical Crystals. Chem. Mater..

[13] Marchenko E.I., Fateev S.A., Petrov A.A., Korolev V.V., Mitrofanov A., Petrov A.V., Goodilin E.A., Tarasov A.B. (2020). Database of Two-Dimensional Hybrid Perovskite Materials: Open-Access Collection of Crystal Structures, Band Gaps, and Atomic Partial Charges Predicted by Machine Learning. Chem. Mater..

[14] Burger S., Ehrenreich M.G., Kieslich G. (2018). Tolerance Factors of Hybrid Organic–Inorganic Perovskites: Recent Improvements and Current State of Research. J. Mater. Chem. A Mater..

[15] McNulty J.A., Lightfoot P. (2021). Structural Chemistry of Layered Lead Halide Perovskites Containing Single Octahedral Layers. IUCrJ.

[16] Ning W., Gao F. (2019). Structural and Functional Diversity in Lead-Free Halide Perovskite Materials. Adv. Mater..

[17] Sani F., Shafie S., Lim H., Musa A. (2018). Advancement on Lead-Free Organic-Inorganic Halide Perovskite Solar Cells: A Review. Materials.

[18] Šenjug P., Dragović J., Kalanj M., Torić F., Rubčić M., Pajić D. (2019). Magnetic Behaviour of (C_2_H_5_NH_3_)_2_CuCl_4_ Type Multiferroic. J. Magn. Magn. Mater..

[19] Han C., Bradford A.J., McNulty J.A., Zhang W., Halasyamani P.S., Slawin A.M.Z., Morrison F.D., Lee S.L., Lightfoot P. (2022). Polarity and Ferromagnetism in Two-Dimensional Hybrid Copper Perovskites with Chlorinated Aromatic Spacers. Chem. Mater..

[20] Han C., McNulty J.A., Bradford A.J., Slawin A.M.Z., Morrison F.D., Lee S.L., Lightfoot P. (2022). Polar Ferromagnet Induced by Fluorine Positioning in Isomeric Layered Copper Halide Perovskites. Inorg. Chem..

[21] Sun B., Liu X.-F., Li X.-Y., Zhang Y., Shao X., Yang D., Zhang H.-L. (2020). Two-Dimensional Perovskite Chiral Ferromagnets. Chem. Mater..

[22] Bloomquist D.R., Willett R.D. (1982). Thermochromic Phase Transitions in Transition Metal Salts. Coord. Chem. Rev..

[23] Bhattacharya R., Sinha Ray M., Dey R., Righi L., Bocelli G., Ghosh A. (2002). Synthesis, Crystal Structure and Thermochromism of Benzimidazolium Tetrachlorocuprate: (C_7_H_7_N_2_)_2_[CuCl_4_]. Polyhedron.

[24] Willett R.D., Haugen J.A., Lebsack J., Morrey J. (1974). Thermochromism in Copper(II) Chlorides. Coordination Geometry Changes in Tetrachlorocuprate(2-)Anions. Inorg. Chem..

[25] Fu H., Jiang C., Luo C., Lin H., Peng H. (2021). A Quasi-Two-Dimensional Copper Based Organic-Inorganic Hybrid Perovskite with Reversible Thermochromism and Ferromagnetism. Eur. J. Inorg. Chem..

[26] Gupta S., Pandey T., Singh A.K. (2016). Suppression of Jahn–Teller Distortions and Origin of Piezochromism and Thermochromism in Cu–Cl Hybrid Perovskite. Inorg. Chem..

[27] Dutta S., Vishnu S.K.D., Som S., Chaurasiya R., Patel D.K., Moovendaran K., Lin C.-C., Chen C.-W., Sankar R. (2022). Segmented Highly Reversible Thermochromic Layered Perovskite [(CH_2_)_2_ (NH_3_)_2_]CuCl_4_ Crystal Coupled with an Inverse Magnetocaloric Effect. ACS Appl. Electron. Mater..

[28] Lufaso M.W., Woodward P.M. (2004). Jahn–Teller Distortions, Cation Ordering and Octahedral Tilting in Perovskites. Acta Crystallogr. B.

[29] Freeman J.H., Smith M.L. (1958). The Preparation of Anhydrous Inorganic Chlorides by Dehydration with Thionyl Chloride. J. Inorg. Nucl. Chem..

[30] Hercouet A., le Corre M. (1988). Triphenylphosphonium Bromide: A Convenient and Quantitative Source of Gaseous Hydrogen Bromide. Synthesis.

[31] (2022). CrysAlisPro.

[32] Sheldrick G.M. (2015). *SHELXT*—Integrated Space-Group and Crystal-Structure Determination. Acta Cryst. A Found Adv..

[33] Sheldrick G.M. (2015). Crystal Structure Refinement with *SHELXL*. Acta Cryst. C Struct. Chem..

[34] Momma K., Izumi F. (2011). *VESTA 3* for Three-Dimensional Visualization of Crystal, Volumetric and Morphology Data. J. Appl. Cryst..

[35] Groom C.R., Bruno I.J., Lightfoot M.P., Ward S.C. (2016). The Cambridge Structural Database. Acta Cryst. B Struct. Sci. Cryst. Eng. Mater..

[36] Evans J.S.O. (2010). Advanced Input Files & Parametric Quantitative Analysis Using Topas. Mater. Sci. Forum..

[37] Cheary R.W., Coelho A. (1992). A Fundamental Parameters Approach to X-Ray Line-Profile Fitting. J. Appl. Cryst..

[38] Harris K.D.M. (2022). Circumventing a Challenging Aspect of Crystal Structure Determination from Powder Diffraction Data. Acta Cryst. B Struct. Sci. Cryst. Eng. Mater..

[39] Nakamoto K. (2008). Infrared and Raman Spectra of Inorganic and Coordination Compounds.

[40] Shannon R.D. (1976). Revised Effective Ionic Radii and Systematic Studies of Interatomic Distances in Halides and Chalcogenides. Acta Crystallogr. Sect. A.

[41] Long G.S., Wei M., Willett R.D. (1997). Crystal Structures and Magnetic Properties of a Novel Layer Perovskite System: (3-Picoliniumylammonium)CuX4 (X = Cl, Br). Inorg. Chem..

[42] Willett R., Place H., Middleton M. (1988). Crystal Structures of Three New Copper(II) Halide Layered Perovskites: Structural, Crystallographic, and Magnetic Correlations. J. Am. Chem. Soc..

[43] Akrout F., Hajlaoui F., Karoui K., Audebrand N., Roisnel T., Zouari N. (2020). Two-Dimensional Copper (II) Halide-Based Hybrid Perovskite Templated by 2-Chloroethylammonium: Crystal Structures, Phase Transitions, Optical and Electrical Properties. J. Solid State Chem..

[44] Takahashi M., Hoshino N., Sambe K., Takeda T., Akutagawa T. (2020). Dynamics of Chiral Cations in Two-Dimensional CuX _4_ and PbX _4_ Perovskites (X = Cl and Br). Inorg. Chem..

[45] Li M., Teng B., Han S., Yang T., Li Y., Liu Y., Zhang X., Liu X., Luo J., Sun Z. (2019). Near-Room-Temperature Tunable Dielectric Response Induced by Dual Phase Transitions in a Lead-Free Hybrid: (C_3_H_8_N)_2_ SbBr_5_. CrystEngComm.

[46] Zhang Z.-X., Su C.-Y., Gao J.-X., Zhang T., Fu D.-W. (2021). Mechanochemistry Enables Optical-Electrical Multifunctional Response and Tunability in Two-Dimensional Hybrid Perovskites. Sci. China Mater..

[47] Han S., Liu X., Zhang J., Ji C., Wu Z., Tao K., Wang Y., Sun Z., Luo J. (2018). Dielectric Phase Transition Triggered by the Order–Disorder Transformation of Cyclopropylamine in a Layered Organic–Inorganic Halide Perovskite. J. Mater. Chem. C Mater..

[48] Yang T., Teng B., Han S., Li M., Xu Z., Li Y., Liu Y., Luo J., Sun Z. (2019). Structural Phase Transition and Dielectric Anisotropy Properties of a Lead-Free Organic–Inorganic Hybrid. Inorg. Chem. Front..

[49] Seeboth A., Ruhmann R., Mühling O. (2010). Thermotropic and Thermochromic Polymer Based Materials for Adaptive Solar Control. Materials.

[50] Zeng Y., Huang X., Huang C., Zhang H., Wang F., Wang Z. (2021). Unprecedented 2D Homochiral Hybrid Lead-Iodide Perovskite Thermochromic Ferroelectrics with Ferroelastic Switching. Angew. Chem. Int. Ed..

[51] Sun B., Liu X., Li X., Cao Y., Yan Z., Fu L., Tang N., Wang Q., Shao X., Yang D. (2020). Reversible Thermochromism and Strong Ferromagnetism in Two-Dimensional Hybrid Perovskites. Angew. Chem. Int. Ed..

[52] Pareja-Rivera C., Solis-Ibarra D. (2021). Reversible and Irreversible Thermochromism in Copper-Based Halide Perovskites. Adv. Opt. Mater..

