# Supplementary material for: Structural Insights into Layered Tetrahalocuprates(II) Based on Small Unsaturated and Cyclic Primary Ammonium Cations
Source: Materials (Basel). 2023 Mar 10;16(6):2236. doi: 10.3390/ma16062236 (PMC10055728; doi:10.3390/ma16062236)
Supplement: Supplementary file 1 [file materials-16-02236-s001.zip › Supporting_Information.pdf]

Structural insights into layered tetrahalocuprates(II) based on small unsaturated and cyclic primary ammonium cations

Edi Topić and Mirta Rubčić*

Department of Chemistry, Faculty of Science, University of Zagreb, Horvatovac 102a, 10000 Zagreb, Croatia
* Correspondence: mirta@chem.pmf.hr

Electronic Supplementary Information

Contents

FTIR spectra of prepared compounds 2
Crystallographic and structural data 8
Rietveld refinement results and TOPAS input files 22
aa_2CuCl_4 22
aa_2CuBr_4 25
$aacn_2CuBr_4$ 29
cpa_2CuBr_4 32
$cpma_2CuBr_4$ 35
Refinements with crystal structure from SCXRD data 39
Thermal analysis 42

FTIR spectra of prepared compounds

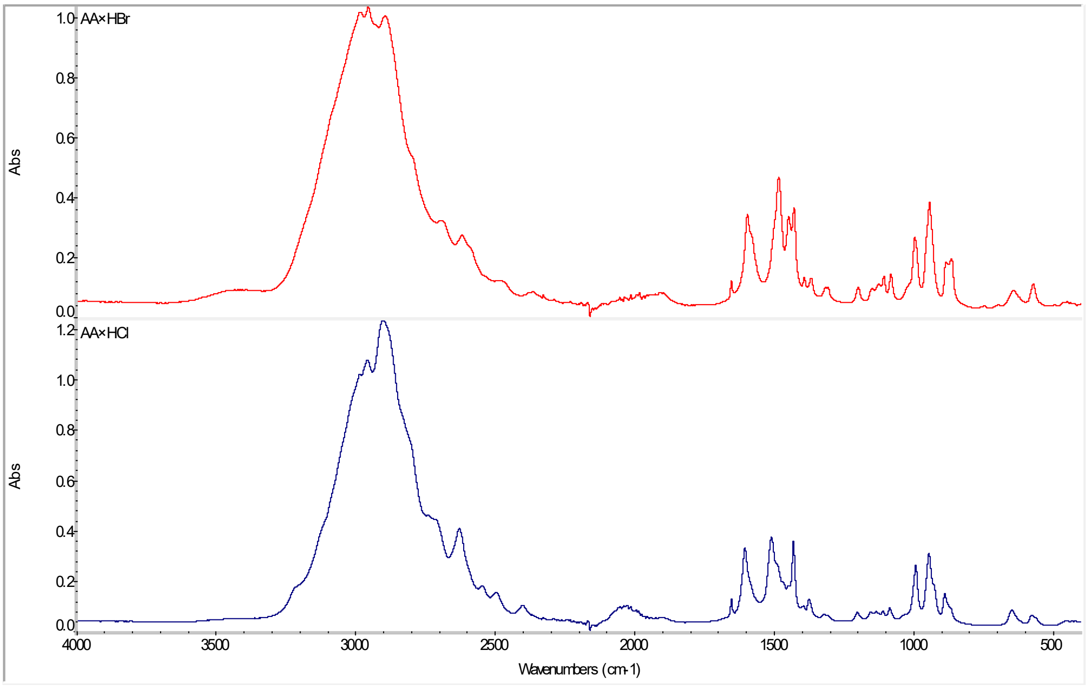

Figure S1. FTIR spectra of **aa×HCl** (bottom) and **aa×HBr** (top). As expected, the spectra are qualitatively similar.

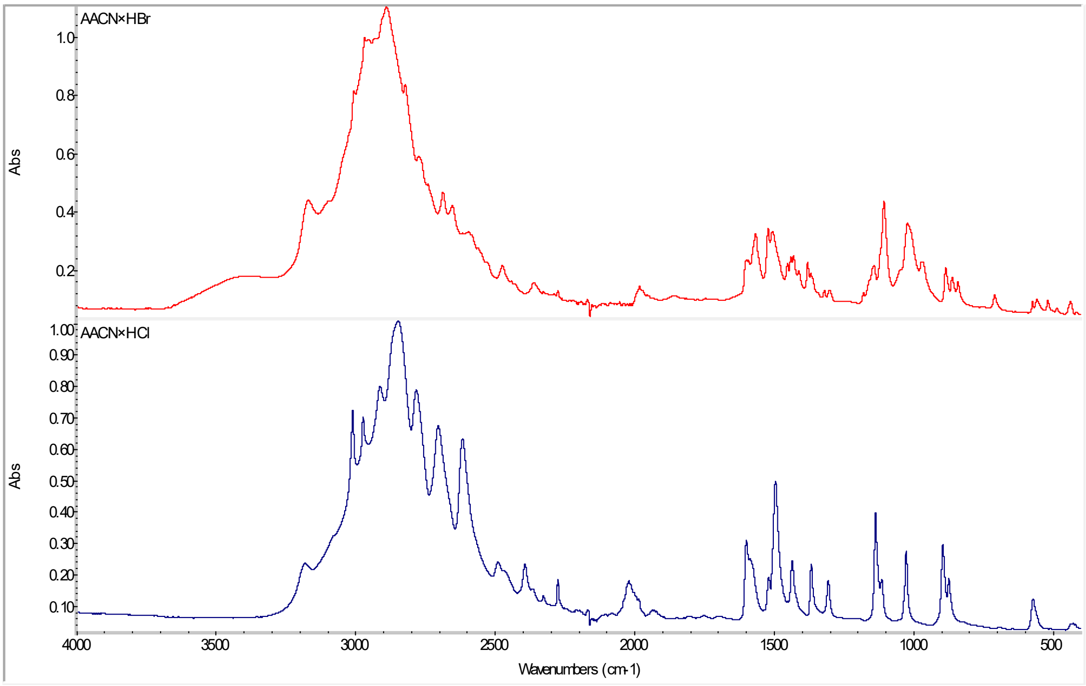

Figure S2. FTIR spectra of **aacn×HCl** (bottom) and **aacn×HBr** (top). As expected, the spectra are qualitatively similar.

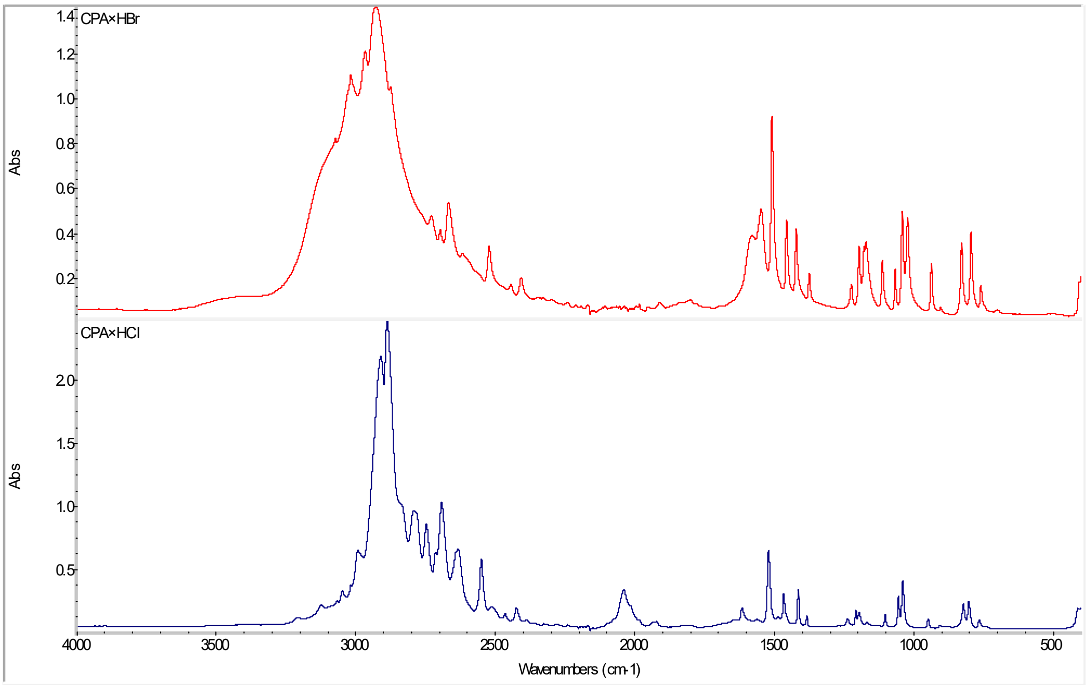

Figure S3. FTIR spectra of **cpa×HCl** (bottom) and **cpa×HBr** (top). There are some differences in C-H and N-H region (2500-3200 cm^{-1}) indicating differences in hydrogen bonding patterns, but the rest of the spectra is qualitatively similar.

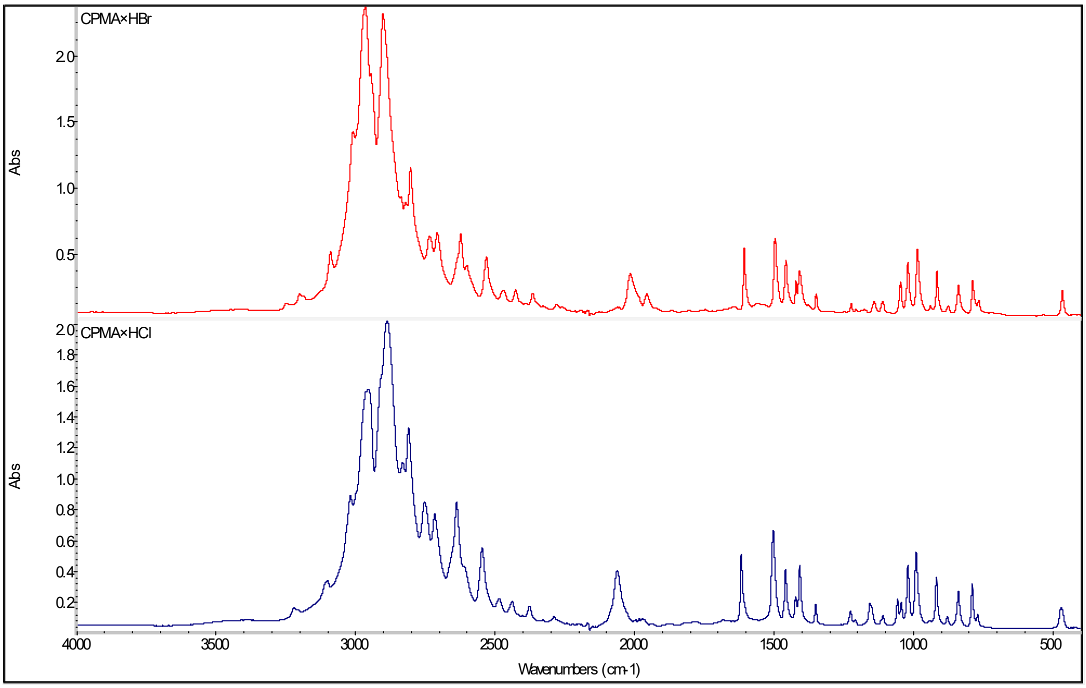

Figure S4. FTIR spectra of **cpma×HCl** (bottom) and **cpma×HBr** (top). As expected, the spectra are qualitatively similar.

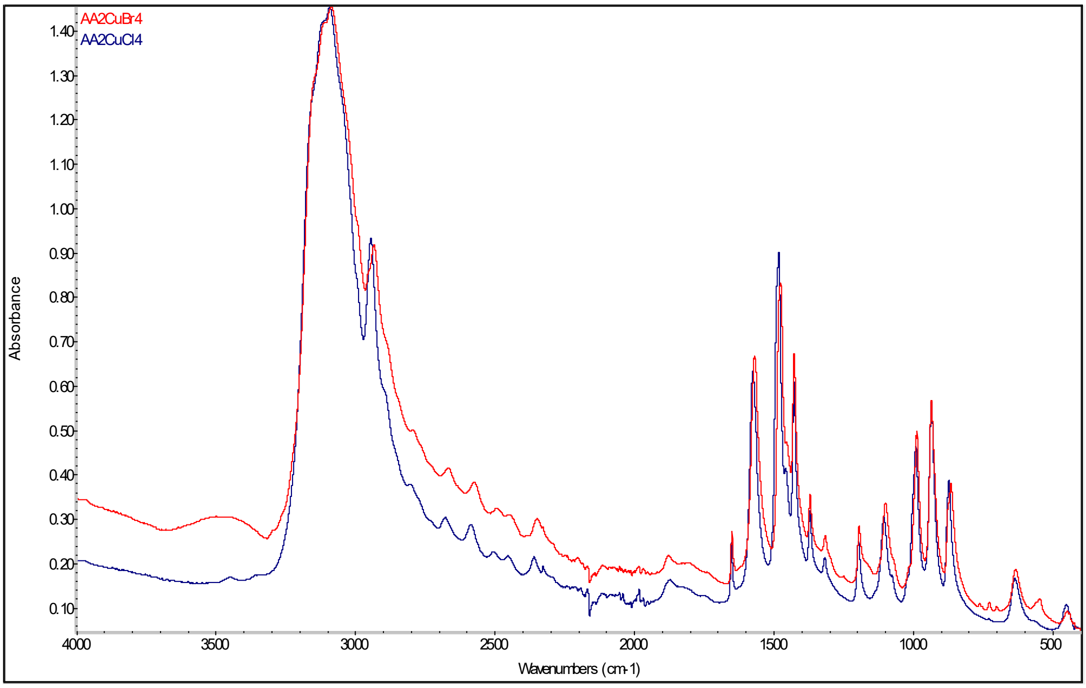

Figure S5. FTIR spectra of $\mathbf{aa_2CuCl_4}$ (blue) and $\mathbf{aa_2CuBr_4}$ (red). As most of the absorption bands originates from the organic cation, the spectra are practically identical.

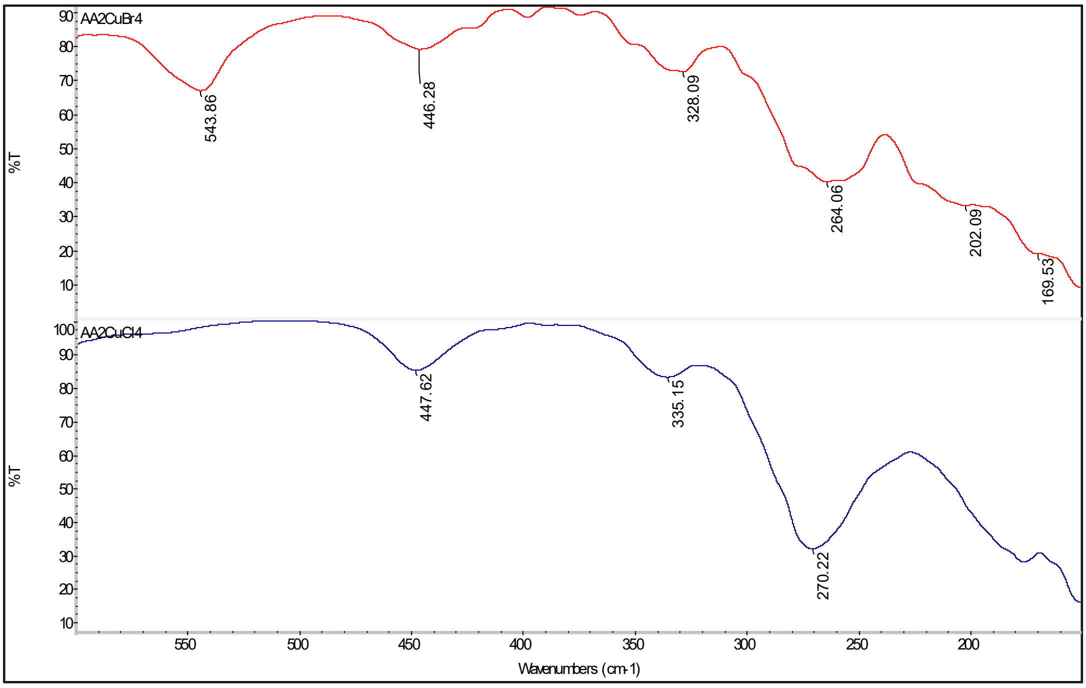

Figure S6. Far-FTIR spectra of $\mathbf{aa_2CuCl_4}$ (bottom) and $\mathbf{aa_2CuBr_4}$ (top). Tetrahalocuprate bands are present as a broad maxima at 270 cm^{-1} for $\mathbf{aa_2CuCl_4}$ and as a weak broad maxima at 202 cm^{-1} for $\mathbf{aa_2CuBr_4}$.

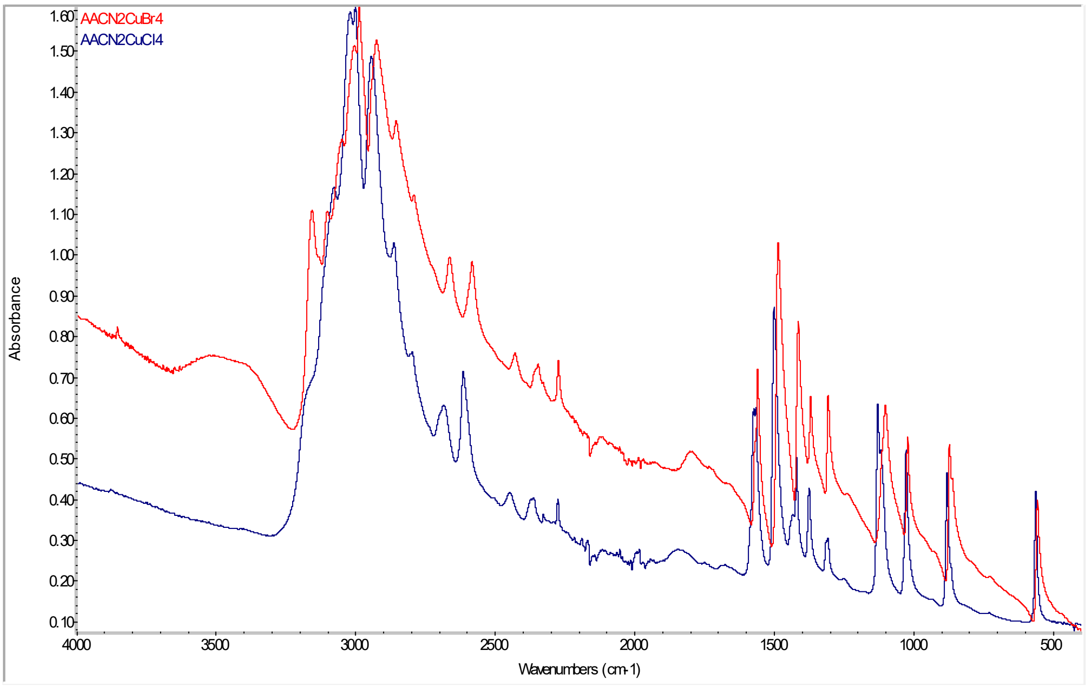

Figure S7. FTIR spectra of **aacn$_2$CuCl$_4$** (blue) and **aacn$_2$CuBr$_4$** (red). As most of the absorption bands originates from the organic cation, the spectra are practically identical, if not slightly shifted.

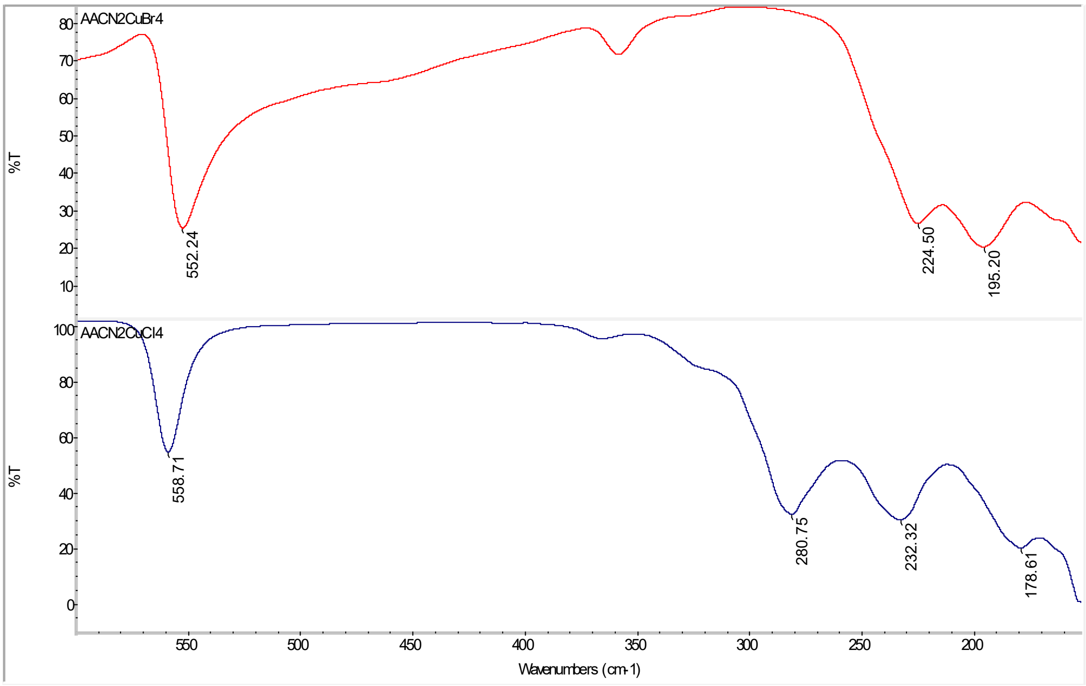

Figure S8. Far-FTIR spectra of **aacn$_2$CuCl$_4$** (bottom) and **aacn$_2$CuBr$_4$** (top). Tetrahalocuprate bands are present as two bands at 280 and 232 cm^{-1} for **aacn$_2$CuCl$_4$** and as two bands at 224 and 195 cm^{-1} for **aacn$_2$CuBr$_4$**.

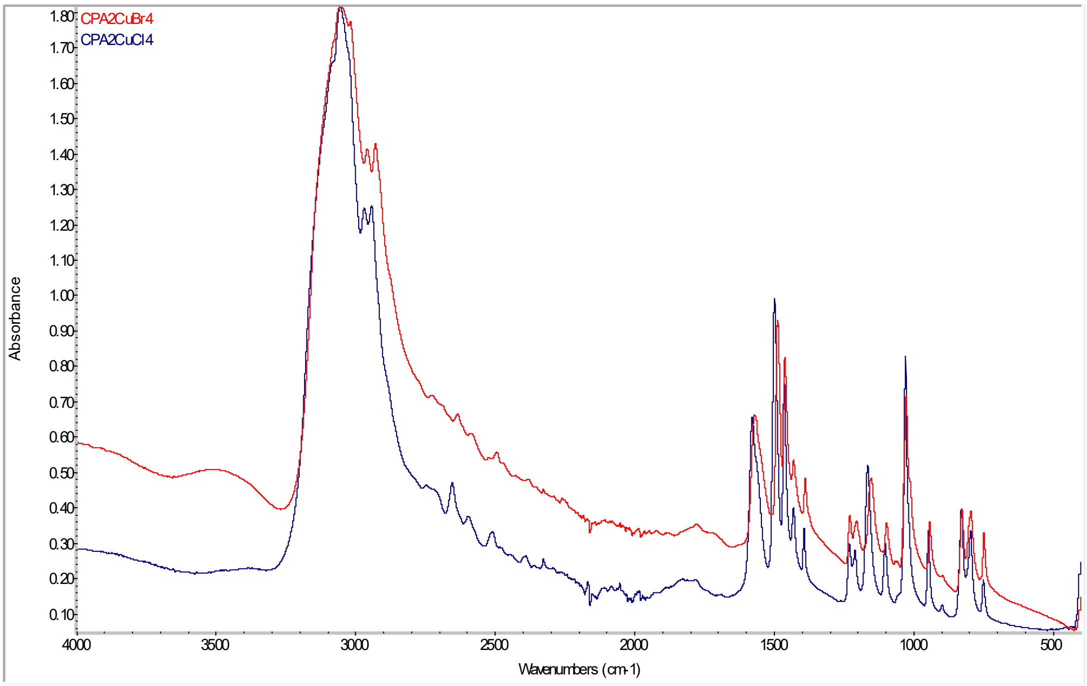

Figure S9. FTIR spectra of **cpa_2CuCl_4** (blue) and **cpa_2CuBr_4** (red). As most of the absorption bands originates from the organic cation, the spectra are practically identical.

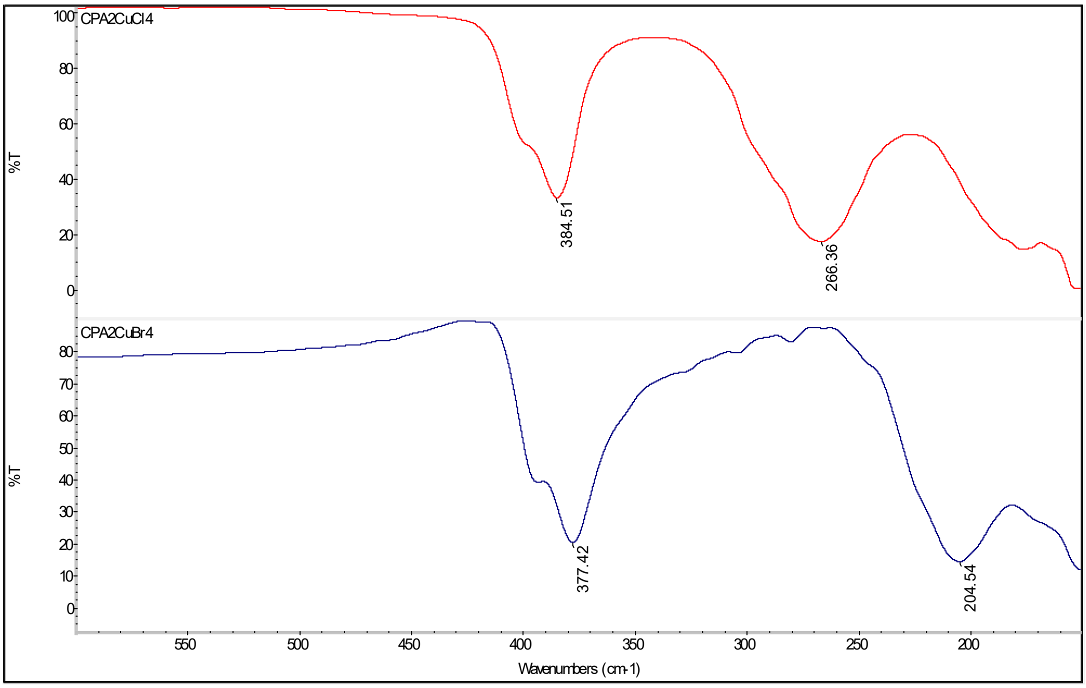

Figure S10. Far-FTIR spectra of **cpa_2CuCl_4** (bottom) and **cpa_2CuBr_4** (top). Tetrahalocuprate bands are present as a broad maxima at 266 cm^{-1} for **cpa_2CuCl_4** and as a broad maxima at 204 cm^{-1} for **cpa_2CuBr_4**.

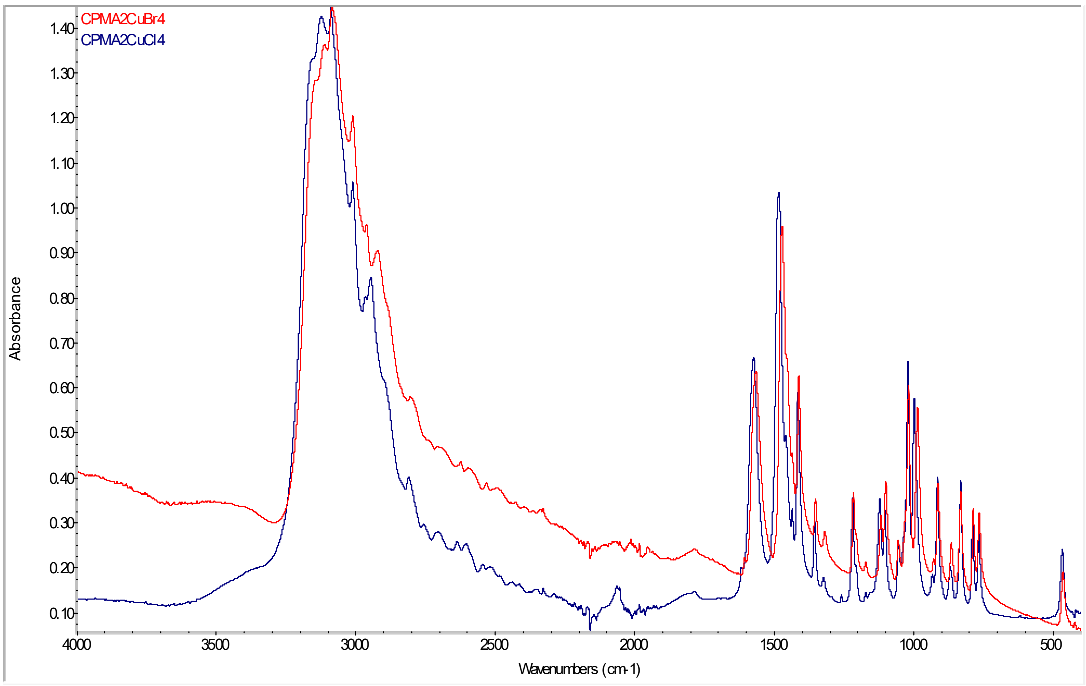

Figure S11. FTIR spectra of **cpma$_2$CuCl$_4$** (blue) and **cpma$_2$CuBr$_4$** (red). As most of the absorption bands originates from the organic cation, the spectra are practically identical, if not slightly shifted.

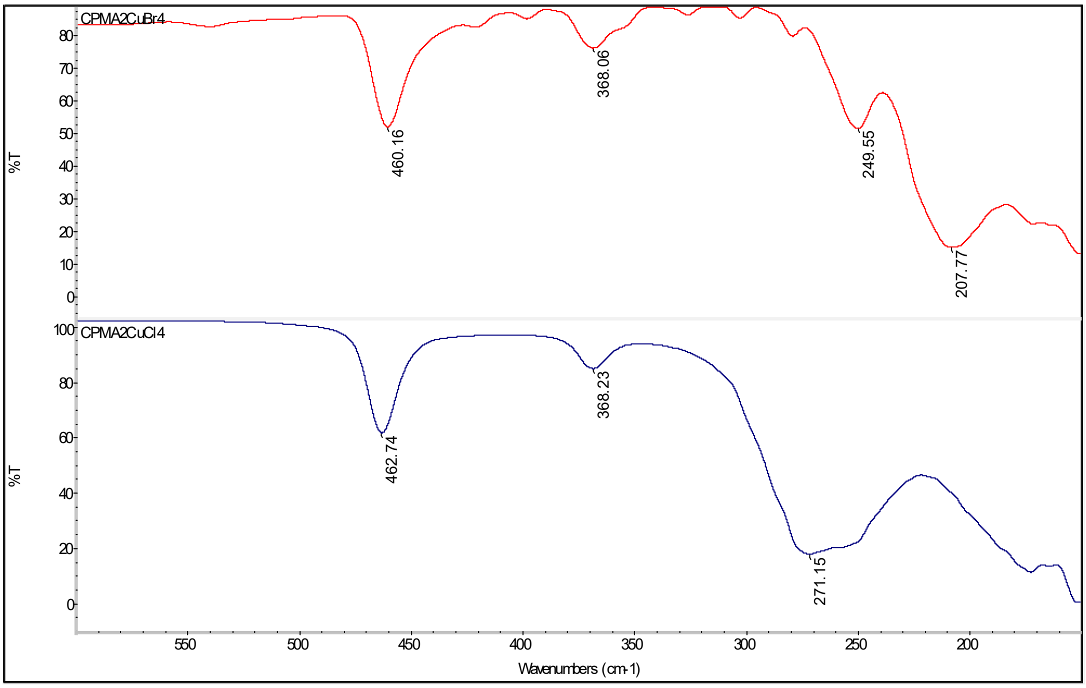

Figure S12. Far-FTIR spectra of **cpma$_2$CuCl$_4$** (bottom) and **cpma$_2$CuBr$_4$** (top). Tetrahalocuprate bands are present as a broad maxima at 271 cm^{-1} for **cpma$_2$CuCl$_4$** and as two bands at 249 and 207 cm^{-1} for **cpma$_2$CuBr$_4$**.

Crystallographic and structural data

Table S1. Experimental and crystallographic data for **cpa_2CuCl_4** and **$cpma_2CuCl_4$** (refined from SCXRD data).

Compound	**cpa_2CuCl_4**	**$cpma_2CuCl_4$**
Empirical formula	$C_6H_{16}Cl_4CuN_2$	$C_8H_{20}Cl_4CuN_2$
M_r	321.55	349.6
T/K	169.99(10)	169.99(10)
Crystal system	monoclinic	orthorhombic
Space group	*C*2/*c*	*Pccn*
a/Å	22.4129(18)	26.7751(6)
b/Å	7.5883(4)	7.6209(2)
c/Å	7.2539(4)	7.1258(2)
α/°	90	90
β/°	93.020(6)	90
γ/°	90	90
V/Å^3	1232.00(14)	1454.02(6)
Z	4	4
ρ_{calc}/g cm^{-3}	1.734	1.597
μ/mm^{-1}	10.171	8.668
F(000)	652	716
Crystal size/mm^3	0.152 × 0.081 × 0.026	0.353 × 0.272 × 0.062
Radiation	Cu Kα (λ = 1.54184)	Cu Kα (λ = 1.54184)
2Θ range/°	7.9 to 161.47	6.602 to 160.856
Index ranges	$-27 \leq h \leq 27$, $-9 \leq k \leq 9$, $-8 \leq l \leq 9$	$-34 \leq h \leq 34$, $-9 \leq k \leq 9$, $-7 \leq l \leq 9$
Reflections collected	2286	16493
Independent reflections	2286 [merged R_{int}, R_{sigma} = 0.0385]	1588 [R_{int} = 0.0857, R_{sigma} = 0.0350]
Data/restraints/ parameters	2286/2/73	1588/0/71
Goodness–of–fit on F^2, S^b	1.127	1.144
Final R and wR^c values [$I \geq 2\sigma(I)$]	R_1 = 0.0685, wR_2 = 0.1924	R_1 = 0.0772, wR_2 = 0.2162
Final R and wR^c values [all data]	R_1 = 0.0823, wR_2 = 0.2129	R_1 = 0.0783, wR_2 = 0.2169

Largest diff. peak/hole / e $Å^{-3}$	1.02/–1.04	2.05/–0.60

[a]$w = 1/[\sigma^2(F_o^2) + (g_1P)^2 + g_2P]$ where $P = (F_o^2 + 2F_c^2)/3$

[b]$S = \{\Sigma[w(F_o^2 - F_c^2)^2]/(N_r - N_p)\}^{1/2}$ where N_r = number of independent reflections, N_p = number of refined parameters.

[c]$R = \Sigma||F_o| - |F_c||/ \Sigma|F_o|$; $wR = \{\Sigma[w(F_o^2 - F_c^2)^2]/\Sigma[w(F_o^2)^2]\}^{1/2}$

Table S2. Crystallographic data for **aacn$_2$CuCl$_4$** (refined from temperature-dependent SCXRD data). All experiments have the common empirical formula ($C_4H_{10}Cl_4CuN_4$), M_r (319.50), crystal system and space group (orthorhombic *Pbca*), crystal size (0.12×0.12×0.03 mm^3) and used the same radiation (Cu Kα).

T/K	a/Å	b/Å	c/Å	V/Å^3	ρ_{calc} g/cm^3	μ/mm^{-1}
169.99(10)	7.30280(10)	7.06850(10)	21.8484(3)	1127.81(3)	1.882	11.168
179.99(10)	7.30850(10)	7.07560(10)	21.8414(2)	1129.46(2)	1.879	11.152
189.99(10)	7.31460(10)	7.08220(10)	21.8363(2)	1131.20(2)	1.876	11.134
200.00(10)	7.32060(10)	7.08740(10)	21.8341(2)	1132.84(2)	1.873	11.118
209.99(10)	7.32720(10)	7.09280(10)	21.8337(2)	1134.71(2)	1.87	11.1
219.99(10)	7.33290(10)	7.09930(10)	21.8313(2)	1136.50(2)	1.867	11.082
229.98(10)	7.33630(10)	7.10490(10)	21.8293(2)	1137.82(2)	1.865	11.07
239.98(10)	7.34060(10)	7.11080(10)	21.8283(2)	1139.38(2)	1.863	11.054
249.97(10)	7.34540(10)	7.11720(10)	21.8303(3)	1141.26(3)	1.859	11.036
260.00(10)	7.34830(10)	7.12320(10)	21.8300(4)	1142.66(3)	1.857	11.023
270.00(10)	7.35070(10)	7.12810(10)	21.8347(4)	1144.06(3)	1.855	11.009
279.99(10)	7.35470(10)	7.13310(10)	21.8437(3)	1145.96(3)	1.852	10.991
289.99(10)	7.35630(10)	7.1398(2)	21.8517(5)	1147.71(4)	1.849	10.974
299.98(10)	7.3598(2)	7.1455(2)	21.8545(5)	1149.32(5)	1.846	10.959
319.98(11)	7.3640(2)	7.1573(2)	21.8482(7)	1151.54(6)	1.843	10.938
340.00(13)	7.3677(2)	7.1645(2)	21.8005(8)	1150.76(6)	1.844	10.945
359.99(16)	7.3611(2)	7.1744(4)	21.6970(9)	1145.85(9)	1.852	10.992
380.00(17)	7.3612(4)	7.1892(5)	21.6531(16)	1145.91(13)	1.852	10.992

Table S3. Crystallographic data for **$aacn_2CuCl_4$** (contd.).

T/K	Reflns.	Ind. reflns.	D/R/P	GooF	Final R [I>=2σ (I)]	Final R [all data]	Largest diff peak/hole / e Å^{-3}
169.99(10)	28583	1174 [R_{int} = 0.0962, R_{sigma} = 0.0245]	1174/0/74	1.09	R_1 = 0.0282, wR_2 = 0.0728	R_1 = 0.0300, wR_2 = 0.0734	0.41/-0.37
179.99(10)	28913	1180 [R_{int} = 0.0970, R_{sigma} = 0.0248]	1180/0/82	1.11	R_1 = 0.0272, wR_2 = 0.0700	R_1 = 0.0285, wR_2 = 0.0707	0.41/-0.36
189.99(10)	29023	1188 [R_{int} = 0.0931, R_{sigma} = 0.0251]	1188/0/71	1.12	R_1 = 0.0264, wR_2 = 0.0647	R_1 = 0.0281, wR_2 = 0.0654	0.39/-0.37
200.00(10)	29106	1188 [R_{int} = 0.0842, R_{sigma} = 0.0213]	1188/0/71	1.1	R_1 = 0.0260, wR_2 = 0.0654	R_1 = 0.0276, wR_2 = 0.0661	0.36/-0.28
209.99(10)	29027	1191 [R_{int} = 0.0922, R_{sigma} = 0.0217]	1191/0/71	1.09	R_1 = 0.0273, wR_2 = 0.0673	R_1 = 0.0290, wR_2 = 0.0679	0.42/-0.32
219.99(10)	29155	1190 [R_{int} = 0.0812, R_{sigma} = 0.0203]	1190/0/74	1.09	R_1 = 0.0268, wR_2 = 0.0630	R_1 = 0.0287, wR_2 = 0.0636	0.32/-0.28
229.98(10)	29194	1187 [R_{int} = 0.0828, R_{sigma} = 0.0199]	1187/0/74	1.08	R_1 = 0.0271, wR_2 = 0.0630	R_1 = 0.0292, wR_2 = 0.0638	0.28/-0.29
239.98(10)	29169	1191 [R_{int} = 0.0820, R_{sigma} = 0.0208]	1191/0/71	1.07	R_1 = 0.0297, wR_2 = 0.0697	R_1 = 0.0324, wR_2 = 0.0709	0.31/-0.29
249.97(10)	29239	1193 [R_{int} = 0.0839, R_{sigma} = 0.0209]	1193/0/71	1.11	R_1 = 0.0306, wR_2 = 0.0726	R_1 = 0.0332, wR_2 = 0.0741	0.34/-0.30
260.00(10)	29202	1195 [R_{int} = 0.0879, R_{sigma} = 0.0220]	1195/0/71	1.11	R_1 = 0.0308, wR_2 = 0.0741	R_1 = 0.0338, wR_2 = 0.0753	0.31/-0.29
270.00(10)	29230	1199 [R_{int} = 0.0875, R_{sigma} = 0.0222]	1199/0/74	1.08	R_1 = 0.0338, wR_2 = 0.0780	R_1 = 0.0364, wR_2 = 0.0794	0.36/-0.35
279.99(10)	22936	1155 [R_{int} = 0.1256, R_{sigma} = 0.0662]	1155/0/62	1.2	R_1 = 0.0463, wR_2 = 0.1145	R_1 = 0.0514, wR_2 = 0.1430	0.73/-0.47
289.99(10)	29315	1192 [R_{int} = 0.0970, R_{sigma} = 0.0252]	1192/0/63	1.08	R_1 = 0.0317, wR_2 = 0.0846	R_1 = 0.0353, wR_2 = 0.0864	0.39/-0.34
299.98(10)	29212	1190 [R_{int} = 0.0922, R_{sigma} = 0.0234]	1190/0/62	1.09	R_1 = 0.0351, wR_2 = 0.0912	R_1 = 0.0398, wR_2 = 0.0937	0.47/-0.41
319.98(11)	28819	1192 [R_{int} = 0.1107, R_{sigma} = 0.0244]	1192/0/62	1.09	R_1 = 0.0487, wR_2 = 0.1263	R_1 = 0.0549, wR_2 = 0.1313	1.15/-0.37
340.00(13)	24076	1189 [R_{int} = 0.1025, R_{sigma} = 0.0309]	1189/0/62	1.05	R_1 = 0.0506, wR_2 = 0.1391	R_1 = 0.0562, wR_2 = 0.1437	1.47/-0.52
359.99(16)	23890	1119 [R_{int} = 0.0989, R_{sigma} = 0.0312]	1119/0/62	1.11	R_1 = 0.0576, wR_2 = 0.1539	R_1 = 0.0638, wR_2 = 0.1641	1.38/-0.75
380.00(17)	21546	1191 [R_{int} = 0.1201, R_{sigma} = 0.0374]	1191/0/62	1.07	R_1 = 0.0596, wR_2 = 0.1659	R_1 = 0.0658, wR_2 = 0.1775	1.27/-0.74

Table S4. Experimental and crystallographic data for **aa_2CuCl_4** and **aa_2CuBr_4** (refined from PXRD data).

Compound	**aa_2CuCl_4**	**aa_2CuBr_4**
Empirical formula	$C_6H_{16}Cl_4CuN_2$	$C_6H_{16}Br_4CuN_2$
M_r	321.56	499.36
Crystal system	monoclinic	monoclinic
Space group	*C*2/*c*	*P*2_1
a/Å	24.1014(7)	12.497(3)
b/Å	7.52712(15)	7.7672(13)
c/Å	7.36310(14)	7.5908(7)
α/°	90	90
β/°	92.270(2)	105.998(13)
γ/°	90	90
V/Å^3	1334.72(5)	708.28(19)
Z	4	2
ρ_{calc}/g cm^{-3}	1.60024(7)	2.3415(6)
μ/mm^{-1}	9.3647(4)	12.556(3)
Radiation	Cu Kα	Mo Kα
2Θ range/°	2.000 to 70.000	2.000 to 35.000
Data/parameters	10358/42	4608/51
Goodness–of–fit, χ^a	4.59	3.47
Final Rp and Rwp^b values/%	4.34, 6.49	4.24, 5.84

$^a\ \chi = \sqrt{\frac{\sum_{i=1}^{N} w_i\left(y_{obs,i}-y_{calc,i}(\boldsymbol{p})\right)^2}{N-P}}$, where w_i is weight (herein equal to $\sqrt{y_{obs,i}}$), $y_{obs,i}$ is the i-th observed intensity, y_{calc_i} i-th calculated intensity, **p** parameter vector, N number of observations and P number of parameters

$^bR_p = \frac{\sum_{i=1}^{N}|y_{obs,i}-y_{calc,i}(\boldsymbol{p})|}{\sum_{i=1}^{N} y_{obs,i}}$, $R_{wp} = \sqrt{\frac{\sum_{i=1}^{N} w_i\left(y_{obs,i}-y_{calc,i}(\boldsymbol{p})\right)^2}{\sum_{i=1}^{N} w_i y_{obs,i}^2}}$

Table S5. Experimental and crystallographic data for **aacn$_2$CuBr$_4$**, **cpa$_2$CuBr$_4$** and **cpma$_2$CuBr$_4$** (refined from PXRD data).

Compound	**aacn$_2$CuBr$_4$**	**cpa$_2$CuBr$_4$**	**cpma$_2$CuBr$_4$**
Empirical formula	$C_4H_{10}Br_4CuN_4$	$C_6Br_4CuN_2$ (hydrogen atoms not modelled)	$C_8H_{20}Br_4CuN_2$
M_r	497.31	483.23	527.41
Crystal system	monoclinic	orthorhombic	orthorhombic
Space group	$P2_1/c$	*Bmab*	*B2cb*
a/Å	10.0769(5)	7.8065(5)	7.7753(4)
b/Å	8.2902(4)	8.0860(5)	7.9773(4)
c/Å	7.5448(3)	21.8629(9)	26.002(2)
α/°	90	90	90
β/°	106.676(4)	90	90
γ/°	90	90	90
V/Å^3	603.77(5)	1380.05(13)	1612.80(18)
Z	4	4	4
ρ_{calc}/g cm^{-3}	2.7355(2)	2.3258(2)	2.1306(2)
μ/mm^{-1}	14.7330(12)	12.8850(12)	11.0329(12)
Radiation	Mo Kα		
2Θ range/°	2.000 to 35.000		
Data/parameters	4608/43	4608/31	4608/52
Goodness–of–fit, χ^a	3.24	3.67	3.88
Final *Rp* and *Rwp*^{b} values/%	3.94, 6.17	4.49, 6.19	5.00, 6.52

[a] $\chi = \sqrt{\frac{\sum_{i=1}^{N} w_i\left(y_{obs,i}-y_{calc,i}(\mathbf{p})\right)^2}{N-P}}$, where w_i is weight (herein equal to $\sqrt{y_{obs,i}}$), $y_{obs,i}$ is the i-th observed intensity, $y_{cal\ i}$ i-th calculated intensity, **p** parameter vector, N number of observations and P number of parameters

[b] $R_p = \frac{\sum_{i=1}^{N}\left|y_{obs,i}-y_{calc,i}(\mathbf{p})\right|}{\sum_{i=1}^{N} y_{obs,i}}$, $R_{wp} = \sqrt{\frac{\sum_{i=1}^{N} w_i\left(y_{obs,i}-y_{calc,i}(\mathbf{p})\right)^2}{\sum_{i=1}^{N} w_i y_{obs,i}^2}}$

Table S6. Selected bond lengths, angles and torsions in the crystal structure of **aa$_2$CuCl$_4$**. Atom numeration is given on the picture below.

bond lengths/Å				
Cu1	Cl1	2.351(4)		
Cu1	Cl2	2.294(3)		
Cu1	Cl1_a	2.933(4)		
N1	C1	1.454(12)		
C1	C2	1.458(12)		
C2	C3	1.31(3)		
bond angles/°				
Cl1	Cu1	Cl2	90.00(13)	
Cl2	Cu1	Cl1_a	90.00(13)	
Cl1	Cu1	Cl1_a	180	
Cl2	Cu1	Cl2_a	180	
Cl1	Cu1	Cl2_a	90.00(13)	
Cl1_a	Cu1	Cl2_a	90.00(13)	
N1	C1	C2	113.7(11)	
C1	C2	C3	120.1(17)	
torsion angles/°				
N1	C1	C2	C3	123(3)
distance between ammonium nitrogen atom and axial chloride ions/Å				
min	2	3	*max*	*avg*
3.23	3.46	3.94	4.35	3.74

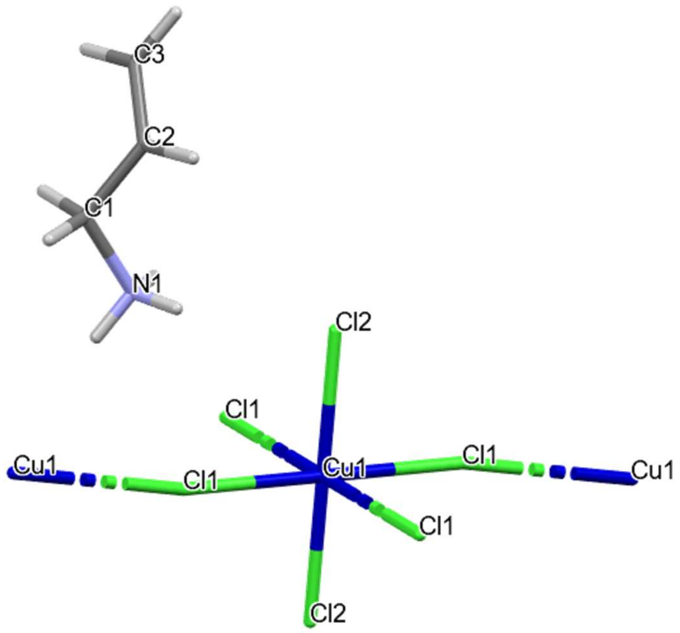

Table S7. Selected bond lengths, angles and torsions in the crystal structure of **aa$_2$CuBr$_4$**. Atom numeration is given on the picture on the side.

bond lengths/Å		
Br1	Cu1	2.46(2)
Br4	Cu1	2.33(3)
Br1	Cu1_b	2.80(2)
Br2	Cu1	2.33(3)
Br3	Cu1	2.45(2)
Br3_b	Cu1	3.17(3)
N1	C1	1.43(16)
C1	C2	1.5(2)
C2	C3	1.3(4)
N11	C11	1.46(15)
C11	C21	1.44(19)
C21	C31	1.3(2)

bond angles/°			
Cu1	Br1	Cu1_b	176.4(13)
Br1	Cu1	Br4	89.9(9)
Br2	Cu1	Br4	179.9(11)
Br3	Cu1	Br1_a	84.7(6)
Br1	Cu1	Br2	90.0(10)
Br1	Cu1	Br1_a	95.3(7)
Br2	Cu1	Br1_a	88.2(9)
Br4	Cu1	Br1_a	91.8(8)
Br1	Cu1	Br3	179.9(14)
Br2	Cu1	Br3	90.0(9)
Br3	Cu1	Br4	90.2(10)
N1	C1	C2	116(14)
C1	C2	C3	119
N11	C11	C21	114(11)
C11	C21	C31	121(17)

torsion angles/°				
N1	C1	C2	C3	77(-1)
N11	C11	C21	C31	-170(-1)
distance between ammonium nitrogen atom and axial chloride ions/Å				
min	2	3	*max*	*avg*
2.95	3.80	3.97	4.94	3.91

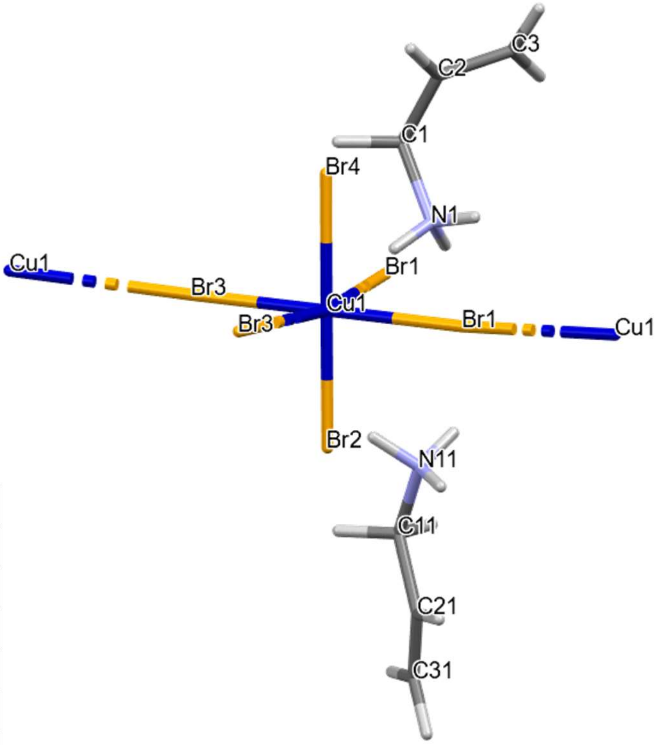

Table S8. Selected bond lengths, angles and torsions in the crystal structure of **aacn$_2$CuCl$_4$**. Atom numeration is given on the picture below.

bond lengths/Å				
Cu1	Cl1	2.3000(7)		
Cu1	Cl2_c	2.2898(5)		
Cu1	Cl2_a	2.8738(6)		
N1	C1	1.457(4)		
N2	C2	1.133(4)		
C1	C2	1.461(5)		
bond angles/°				
Cl1	Cu1	Cl2	89.86(2)	
Cl1	Cu1	Cl2_c	90.15(2)	
Cl1	Cu1	Cl2_a	93.04(2)	
Cl1	Cu1	Cl2_e	86.96(2)	
Cl1	Cu1	Cl1_c	180	
Cl2	Cu1	Cl2_a	91.66(2)	
N1	C1	C2	111.8(3)	
N2	C2	C1	179.5(4)	
torsion angles undefined				
distance between ammonium nitrogen atom and axial chloride ions/Å				
min	2	3	*max*	*avg*
3.23	3.36	3.77	4.14	3.63

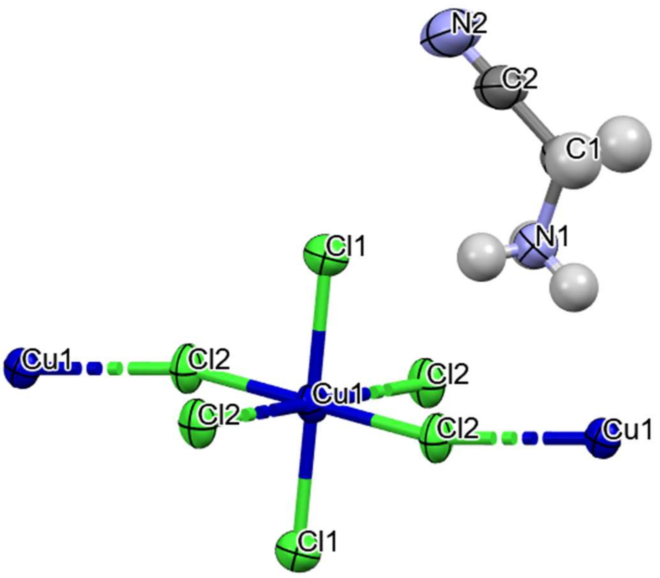

Table S9. Selected bond lengths, angles and torsions in the crystal structure of **$aacn_2CuBr_4$**. Atom numeration is given on the picture below.

bond lengths/Å				
Br1	Cu1	2.490(5)		
Br2	Cu1	2.428(6)		
Br1_a	Cu1	3.198(6)		
N1	C1	1.2(2)		
N2	C2	1.48(17)		
C1	C2	1.46(19)		
bond angles/°				
Br1	Cu1	Br2	89.2(3)	
Br2	Cu1	Br1_a	90.8(3)	
Br1	Cu1	Br1_a	180	
Br2	Cu1	Br2_a	180	
Br1	Cu1	Br2_a	90.8(3)	
Br1_a	Cu1	Br2_a	89.2(3)	
N1	C1	C2	179(19)	
N2	C2	C1	110(7)	
torsion angles undefined				
distance between ammonium nitrogen atom and axial chloride ions/Å				
min	2	3	*max*	*avg*
3.40	3.55	4.04	4.91	3.98

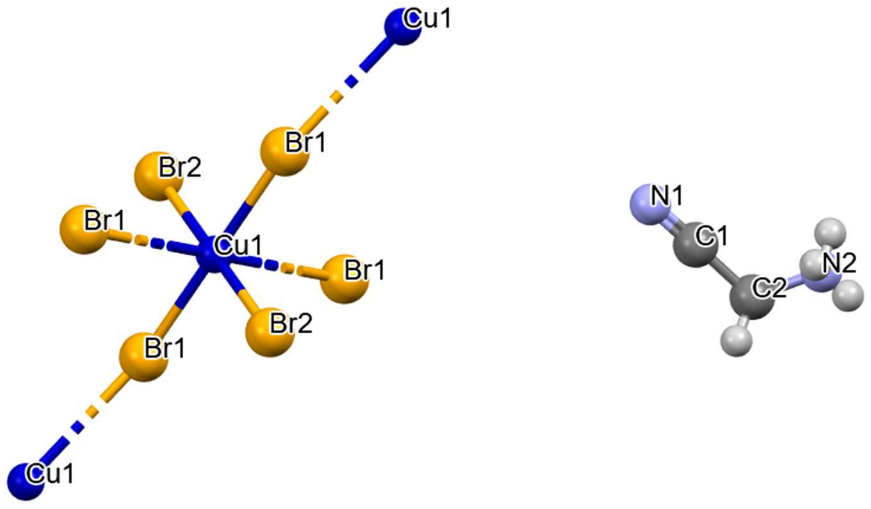

Table S10. Selected bond lengths, angles and torsions in the crystal structure of **cpa$_2$CuCl$_4$**. Atom numeration is given on the picture below.

bond lengths/Å				
Cu1	Cl1	2.2936(15)		
Cu1	Cl2	2.2884(16)		
Cu1	Cl1_a	3.0046(15)		
N1	C1	1.436(12)		
C1	C2	1.422(14)		
C1	C3	1.285(18)		
C1	C3A	1.21(2)		
C2	C3	1.486(16)		
C2	C3A	1.48(2)		
bond angles/°				
Cl1	Cu1	Cl2	89.64(5)	
Cl2	Cu1	Cl1_a	90.37(5)	
Cl1	Cu1	Cl1_a	180	
Cl2	Cu1	Cl2_a	180	
Cl1	Cu1	Cl2_a	90.37(5)	
Cl1_a	Cu1	Cl2_a	89.64(5)	
N1	C1	C3	131.8(12)	
C2	C1	C3A	68.0(12)	
C1	C3	C2	61.2(8)	
N1	C1	C2	123.6(10)	
C2	C1	C3	66.4(9)	
C1	C2	C3A	49.1(11)	
N1	C1	C3A	147.0(17)	
C1	C2	C3	52.4(8)	
C1	C3A	C2	62.9(12)	
torsion angles/°				
N1	C1	C2	C3	125.7(15)
N1	C1	C3	C2	–114.8(16)
distance between ammonium nitrogen atom and axial chloride ions/Å				
min	2	3	*max*	*avg*
3.21	3.41	3.85	4.40	3.71

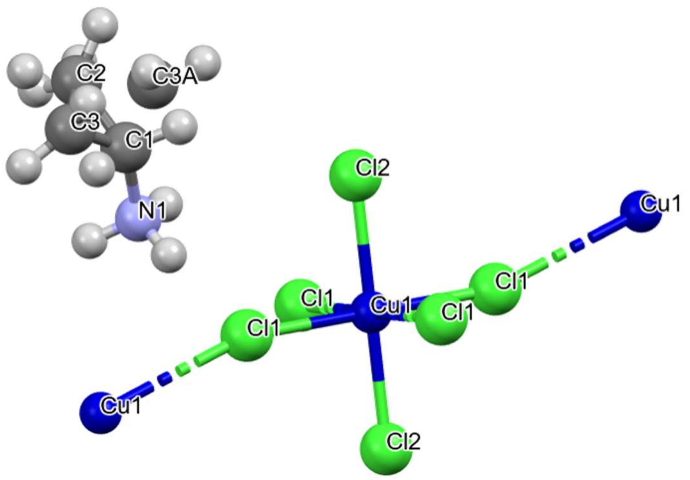

Table S11. Selected bond lengths, angles and torsions in the crystal structure of **cpa$_2$CuBr$_4$**. Atom numeration is given on the picture below. Atoms marked by asterisk (*) are in disorder.

bond lengths/Å				
*Br1	Cu1	2.437(4)		
*Br2	Cu1	2.47(5)		
*N1	*C1	1.50(4)		
*C1	*C2	1.50(7)		
*C1	*C3	1.50(8)		
*C2	*C3	1.51(10)		
bond angles/°				
*Br1	Cu1	*Br2	90.0(3)	
*Br1	Cu1	*Br1_b	180	
*Br1	Cu1	*Br2_c	84.6(3)	
*Br1	Cu1	*Br1_a	172.4(3)	
*Br1	Cu1	*Br2_b	90.0(3)	
*Br2	Cu1	*Br2_a	90.5(15)	
*Br1	Cu1	*Br2_a	95.4(3)	
*Br1	Cu1	*Br1_c	7.6(3)	
*N1	*C1	*C2	109(3)	
*C1	*C2	*C3	60(4)	
*N1	*C1	*C3	111(5)	
*C1	*C3	*C2	60(4)	
*C2	*C1	*C3	61(4)	
torsion angles/°				
*N1	*C1	*C2	*C3	-104(5)
*N1	*C1	*C3	*C2	101(4)
distance between ammonium nitrogen atom and axial chloride ions/Å				
min	2	3	*max*	*avg*
3.69	3.82	4.03	4.41	3.99

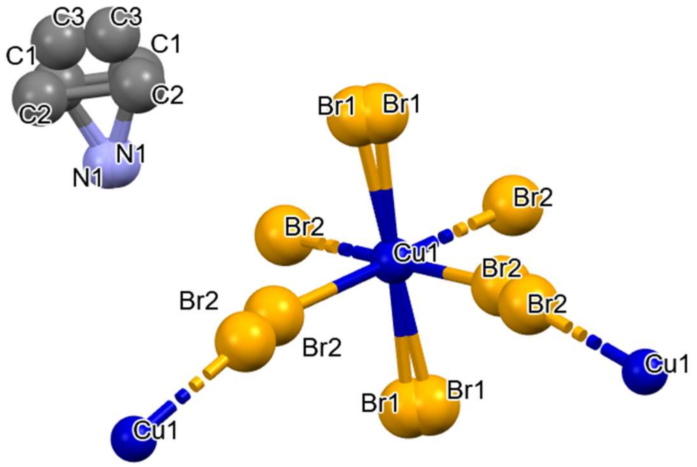

Table S12. Selected bond lengths, angles and torsions in the crystal structure of **cpma$_2$CuCl$_4$**. Atom numeration is given on the picture below.

bond lengths/Å		
Cu1	Cl1	2.2810(13)
Cu1	Cl2	2.3133(16)
Cu1	Cl1_a	2.9790(13)
N1	C1	1.466(9)
C1	C2	1.483(11)
C2	C3	1.493(14)
C2	C4	1.495(12)
C3	C4	1.493(14)

bond angles/°			
Cl1	Cu1	Cl2	90.67(5)
Cl2	Cu1	Cl1_a	89.33(5)
N1	C1	C2	112.6(6)
C3	C2	C4	60.0(7)
Cl1	Cu1	Cl1_a	180
Cl2	Cu1	Cl2_a	180
C1	C2	C3	116.2(8)
C2	C3	C4	60.1(6)
Cl1	Cu1	Cl2_a	89.33(5)
Cl1_a	Cu1	Cl2_a	90.67(5)
C1	C2	C4	118.9(8)
C2	C4	C3	59.9(7)

torsion angles/°				
N1	C1	C2	C3	80.6(9)
N1	C1	C2	C4	149.1(7)
C1	C2	C3	C4	109.8(9)
C1	C2	C4	C3	-105.2(10)

distance between ammonium nitrogen atom and axial chloride ions/Å				
min	2	3	*max*	*avg*
3.26	3.35	3.80	4.38	3.70

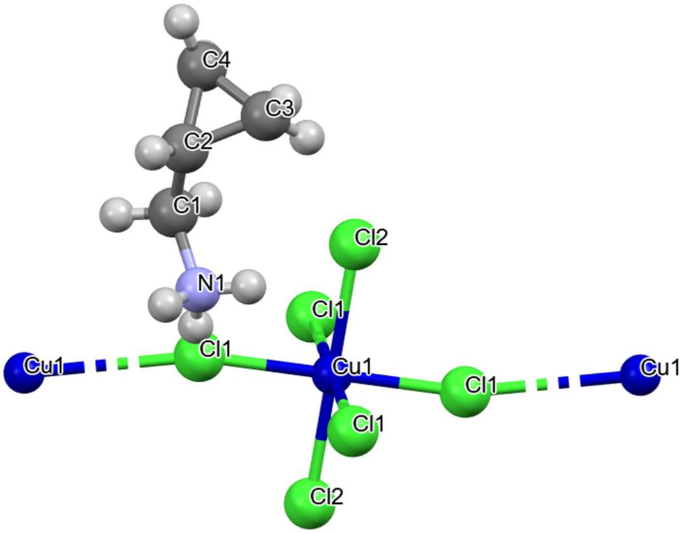

Table S13. Selected bond lengths, angles and torsions in the crystal structure of **$cpma_2CuBr_4$**. Atom numeration is given on the picture below.

bond lengths/Å				
Br1	Cu1	2.400(8)		
Br2	Cu1	2.46(9)		
Br2_a	Cu1	3.18(9)		
N1	C1	1.46(6)		
C3	C4	1.50(18)		
C1	C2	1.48(9)		
C2	C3	1.49(15)		
C2	C4	1.50(16)		
bond angles/°				
Br1	Cu1	Br2	90.0(5)	
Br2	Cu1	Br1_a	98.2(6)	
Br1	Cu1	Br1_a	168.1(4)	
Br2	Cu1	Br2_a	94(3)	
Br1	Cu1	Br2_a	98.2(6)	
Br1_a	Cu1	Br2_a	90.0(5)	
N1	C1	C2	111(6)	
C1	C2	C3	117(7)	
C1	C2	C4	117(8)	
C2	C4	C3	60(7)	
C2	C3	C4	60(8)	
C3	C2	C4	60(8)	
torsion angles/°				
N1	C1	C2	C3	56(11)
N1	C1	C2	C4	125(9)
C1	C2	C3	C4	107(9)
C1	C2	C4	C3	-108(9)
distance between ammonium nitrogen atom and axial chloride ions/Å				
min	2	3	*max*	*avg*
3.66	3.75	4.17	4.25	3.96

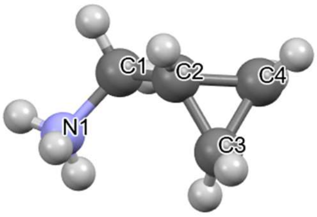

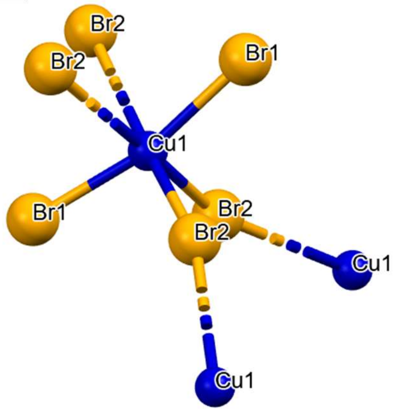

Table S14. Selected bond lengths, angles and torsions in the crystal structure of **$cpma_2CuBr_4$**. Atom numeration is given on the picture below.

d(Cu-Cl_{eq2})/Å	d(Cu-Cl_{ax})/Å	d(Cu-Cl_{eq2})/Å	**Δ**/%
2.2850(4)	2.3062(4)	2.8339(4)	1.0524(13)
2.2851(4)	2.3054(4)	2.8384(4)	1.0702(13)
2.2852(4)	2.3049(4)	2.8430(4)	1.0879(13)
2.2854(4)	2.3045(4)	2.8468(4)	1.1023(14)
2.2858(4)	2.3039(4)	2.8507(4)	1.1171(14)
2.2861(4)	2.3034(4)	2.8545(4)	1.1316(14)
2.2859(4)	2.3030(4)	2.8576(4)	1.1446(14)
2.2864(5)	2.3015(4)	2.8605(5)	1.1577(17)
2.2873(5)	2.3017(4)	2.8632(5)	1.1653(17)
2.2875(5)	2.3008(4)	2.8659(5)	1.1771(17)
2.2879(5)	2.3003(4)	2.8675(5)	1.1834(17)
2.2894(5)	2.3001(4)	2.8714(5)	1.1950(17)
2.2898(5)	2.3000(7)	2.8738(6)	1.203(2)
2.2909(8)	2.2991(9)	2.8776(8)	1.217(3)
2.2907(8)	2.2968(9)	2.8803(8)	1.234(3)
2.2888(11)	2.2920(13)	2.8805(11)	1.251(4)
2.2898(11)	2.290(13)	2.8833(12)	1.264(4)

Rietveld refinement results and TOPAS input files

aa_2CuCl_4

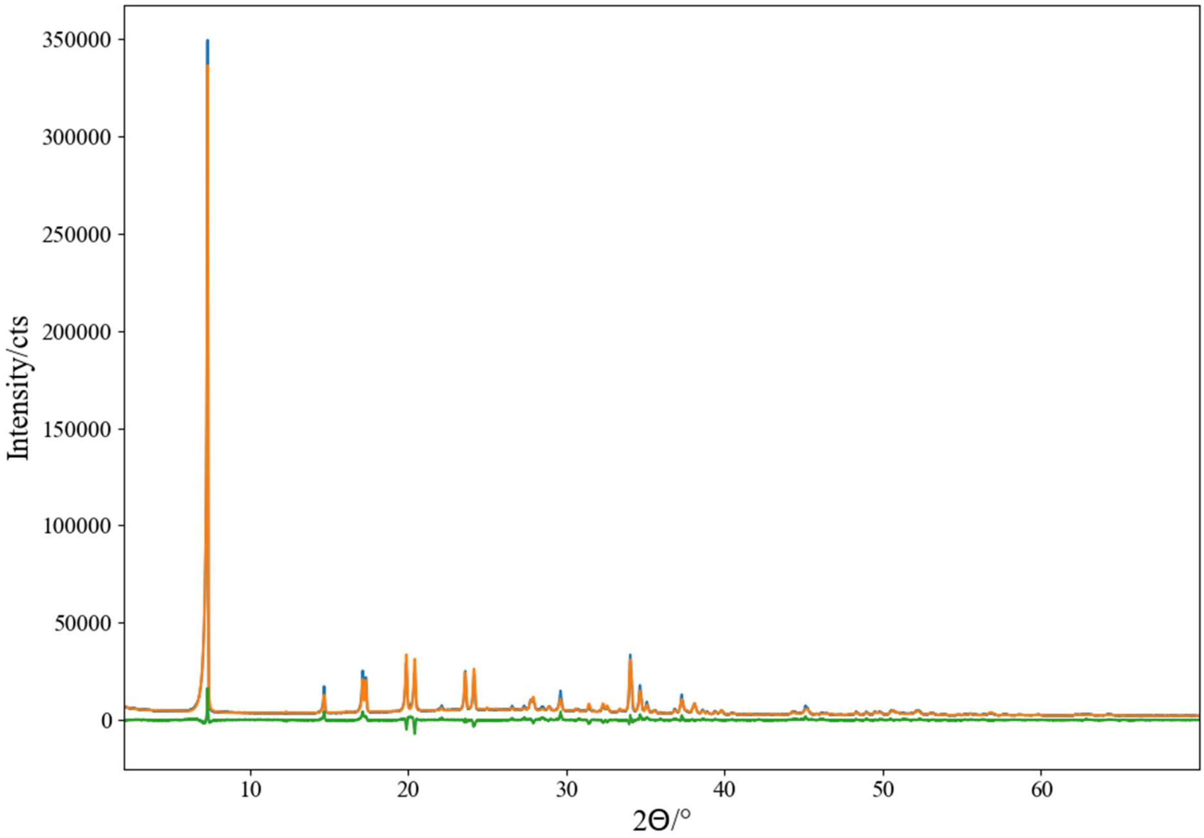

Figure S13. PXRD data for **aa_2CuCl_4** (blue), calculated pattern (orange) and difference between observed and calculated pattern (green).

```
xdd aa2cucl4.xy

'Auto_T(10)

macro rotrans {@}

do_errors

iters 5

       r_wp  6.48712735 r_exp  1.41239667 r_p  4.34550665 r_wp_dash  13.8361246 r_p_dash
13.6258063 r_exp_dash  3.01244222 weighted_Durbin_Watson  0.105724047 gof  4.59299253

   lam

      ymin_on_ymax 0.0001

      la 0.66050 lo 1.540598  lh 0.5

      la 0.33950 lo 1.544426  lh 0.5

       bkg  @  3486.18135`_4.59098099 -1668.1724`_7.38340701  84.5439598`_7.02893062 -
40.8421453`_6.67106647  462.008439`_6.34879313 -625.489019`_6.27499905  234.712707`_6.11572235
149.696503`_5.98637834 -121.896043`_5.76068825 -104.989029`_5.7634446  151.151396`_5.58692193
-45.9410744`_5.5345654  46.4415792`_5.29791197 -43.0781029`_5.10351641  41.1663361`_4.69575019
-50.2477929`_4.50670915

       x_calculation_step 0.005
```

```
Radius(240)

LP_Factor(0)

Zero_Error(@,-0.01634`_0.00025)

   xdd_out "aa2cucl4.txt" load out_record out_fmt out_eqn

{

  " %11.6f  " = X;

 " %11.6f  " = Yobs;

 " %11.6f  " = Ycalc;

 " %11.6f\n" = Yobs-Ycalc;

}

Full_Axial_Model(12, 15, 12,   2.98935_0.17532,  7.67760_0.64523)


str

       phase_name "aa2CuCl4"

       space_group "C2/c"

       a  @  24.101374`_0.000713

       b  @  7.527117`_0.000152

       c  @  7.363098`_0.000141

       be @  92.26955`_0.00217

       CS_G(@, 262.39647`_2.62288)

       Strain_L(@, 0.16013`_0.00310)

       Strain_G(@, 0.23372`_0.00501)

       prm beq3  5.79370`_0.07096 min 1 max 10

       scale @  0.000841325554`_3.425e-006

       site           Cu1     x   0.25000`_0.00000   y  0.25000`_0.00000   z
0.00000`_0.00000              occ Cu  1        beq =beq3;

       site           Cl1     x   0.25935`_0.00013   y  0.47029`_0.00063   z       -
0.22310`_0.00065              occ Cl  1    beq =beq3;

       site           Cl2     x   0.34469`_0.00012   y  0.21903`_0.00066   z
0.02270`_0.00037              occ Cl         1        beq =beq3;

       site           C3      x   0.03092`_0.00035   y  0.80578`_0.00258   z
0.04008`_0.00572              occ C   1 beq =beq3;

       site           C1             x   0.12492`_0.00029   y  0.81737`_0.00165   z
 -0.05566`_0.00213            occ C   1 beq =beq3;

       site           C2      x   0.07066`_0.00025   y  0.73152`_0.00215   z       -
0.04958`_0.00399              occ C          1 beq =beq3;

       site           N1      x   0.17051`_0.00017   y  0.70620`_0.00116   z
0.01188`_0.00084              occ N          1        beq =beq3;

       site           H1             x  0.16753`_0.00268    y      0.60227`_0.00481
z      -0.03604`_0.01212      occ H   1      beq =beq3*1.5;

       site           H2             x  0.20154`_0.00026    y      0.75307`_0.00800
z      -0.01643`_0.01376      occ H   1      beq =beq3*1.5;

       site           H3             x  0.16947`_0.00286    y      0.69737`_0.01267
z      0.12815`_0.00184       occ H   1      beq =beq3*1.5;
```

```
        site          H4             x  0.13196`_0.00071   y      0.84889`_0.00287
z      -0.18608`_0.00217      occ H   1      beq =beq3*1.5;

        site          H5             x  0.12444`_0.00039   y      0.92808`_0.00135
z      0.02176`_0.00309       occ H   1      beq =beq3*1.5;

        site          H6             x  0.06380`_0.00054   y      0.61491`_0.00280
z      -0.11451`_0.00494      occ H   1      beq =beq3*1.5;

        site          H7             x  0.03778`_0.00069   y      0.92239`_0.00298
z      0.10502`_0.00613       occ H   1      beq =beq3*1.5;

        site          H8             x -0.00659`_0.00033   y      0.74643`_0.00306
z      0.04429`_0.00726       occ H   1      beq =beq3*1.5;

        normalize_FCs

        rigid

               prm CuCl1  2.35118`_0.00474_LIMIT_MAX_2.4 min 2.20 max 2.4

               prm CuCl2  2.29353`_0.00294 min 2.2 max 2.4


               point_for_site Cu1 ux 0 uy 0 uz 0

               point_for_site Cl1 ux =CuCl1;  uy 0 uz 0

               point_for_site Cl2 ux 0 uy =CuCl2;  uz 0

               rotate rotrans  357.30365`_0.09504 qa 1

               rotate rotrans  133.10484`_0.11653 qb 1

               rotate rotrans  262.26748`_0.10565 qc 1

               translate ta 0.25 tb 0.25 tc 0

               prm kut1  47.49648`_8.24535_LIMIT_MIN_0 min 0 max 120

        rigid

               z_matrix N1

               z_matrix C1 N1 1.453

               z_matrix C2 C1 1.461 N1 113.6

               z_matrix C3 C2 1.31 C1 120 N1 @  122.90657`_0.73315

               z_matrix H1 N1 0.86 C1 109.5 C2 =kut1;

               z_matrix H2 N1 0.86 C1 109.5 C2 =kut1+120;

               z_matrix H3 N1 0.86 C1 109.5 C2 =kut1+240;

               z_matrix H4 C1 1.01 C2 108.5 N1 120

               z_matrix H5 C1 1.01 C2 108.5 N1 -120

               z_matrix H6 C2 1.01 C3 120 C1 -180

               z_matrix H7 C3 1.01 C2 120 C1 0

               z_matrix H8 C3 1.01 C2 120 C1 180

               rotate rotrans  108.62379`_0.61646 qa 1

               rotate rotrans  345.69211`_0.81851 qb 1

               rotate rotrans  228.66159`_0.24698 qc 1

               translate ta rotrans  0.17051`_0.00017 tb rotrans  0.70620`_0.00116 tc
rotrans  0.01188`_0.00084
```

aa_2CuBr_4

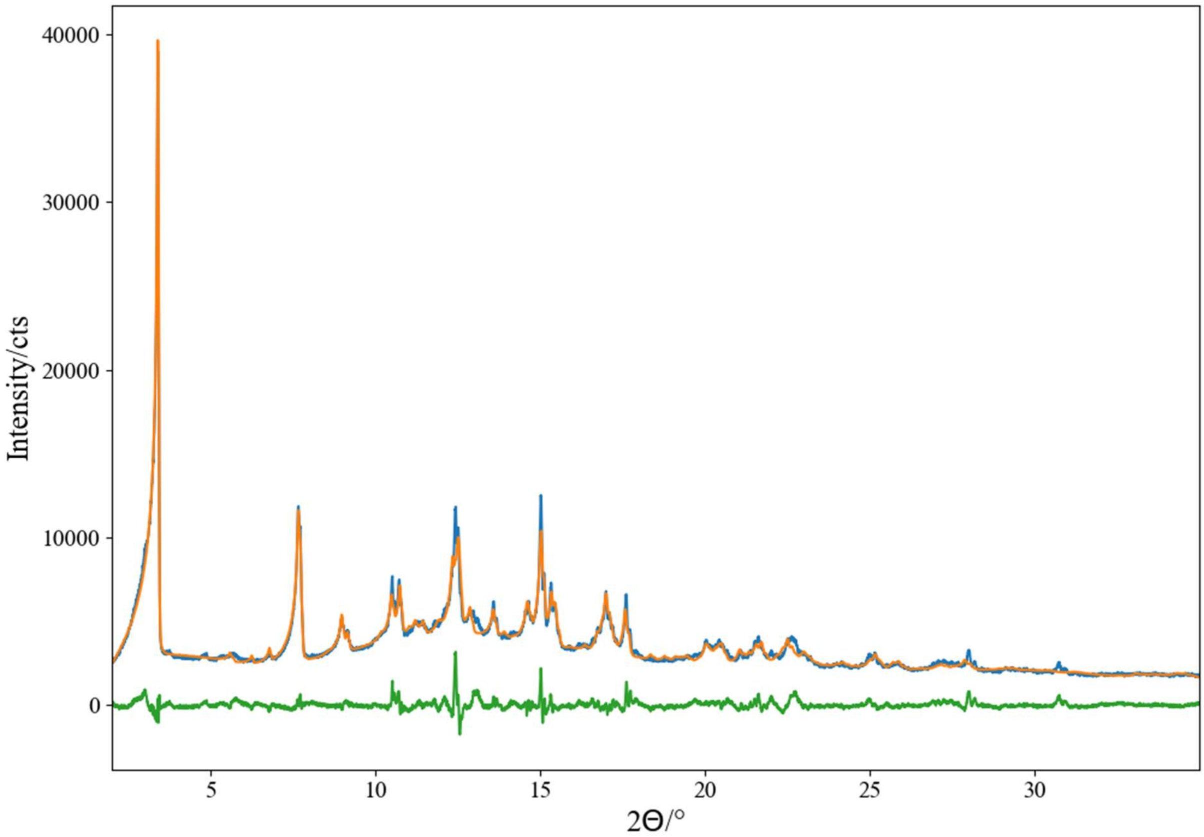

Figure S14. PXRD data for $\mathbf{aa_2CuBr_4}$ (blue), calculated pattern (orange) and difference between observed and calculated pattern (green).

```
xdd aa2cubr4.xy

iters 1000

'Auto_T(50)

do_errors

macro rotrans { @ }

        r_wp  5.84359227 r_exp  1.68041644 r_p  4.24524614 r_wp_dash  17.1195536 r_p_dash  17.0901591 r_exp_dash  4.92299563 weighted_Durbin_Watson  0.203245305 gof  3.47746674

        bkg @  2422.82553`_6.86073751 -488.880305`_8.34475864 -596.533717`_8.62603976 464.943279`_7.19327559 -62.3069883`_6.98650147 -154.037217`_6.47413595  8.39063793`_6.24733384 264.40059`_6.11691803 -301.762824`_6.27887818  134.787703`_6.86485886  41.4749531`_6.10894782 -117.902922`_6.08654077

   lam ymin_on_ymax 0.0001

   Lam_recs

   {

      0.6533  0.709300  0.2695

      0.3467  0.713574  0.2795

   }

   Radius(240)
```

```
       Zero_Error(@, 0.02402`_0.00074)
       LP_Factor(0)
       Full_Axial_Model(12, 15, 12,   43.31400_LIMIT_MIN_0.0001,   4.31758)
       x_calculation_step 0.01
                  xdd_out "aa2cubr4.txt" load out_record out_fmt out_eqn
   {
        " %11.6f  " = X;
       " %11.6f  " = Yobs;
       " %11.6f  " = Ycalc;
       " %11.6f\n" = Yobs-Ycalc;
   }
       str
       a  @  12.497126`_0.002507
       b  @  7.767166`_0.001068
       c  @  7.590787`_0.000729
       be @  105.99829`_0.01349
       space_group "P21"
              CS_G(@, 132.16515`_4.14770)
              Strain_L(@, 0.34327`_0.01582)
              scale @  8.33709585e-005`_0.0006767
              prm beq1  8.17460`_0.35629 min 1 max 10
              site Cu1 x  0.50588`_0.00160 y  0.00177`_0.00000 z  0.73580`_0.00173 occ Cu 1
beq =beq1;
              site Br1 x  0.50725`_0.00218 y  0.23394`_0.00199 z  0.51630`_0.00273 occ Br 1
beq =beq1;
              site Br2 x  0.69878`_0.00198 y -0.01731`_0.00326 z  0.80526`_0.00243 occ Br 1
beq =beq1;
              site Br3 x  0.50451`_0.00218 y -0.23040`_0.00199 z  0.95531`_0.00273 occ Br 1
beq =beq1;
              site Br4 x  0.31299`_0.00198 y  0.02085`_0.00326 z  0.66634`_0.00243 occ Br 1
beq =beq1;

              prm vez1  2.45846`_0.01620_LIMIT_MAX_2.5 min 2.3 max 2.5
              prm vez2  2.32605`_0.01386_LIMIT_MIN_2.3 min 2.3 max 2.5
              'prm kut1  84.12498 min 60 max 90
              'prm kut2  66.95192 min 60 max 90
              rigid
              point_for_site Cu1 ux 0 uy 0 uz 0
              point_for_site Br1 ux =vez1; uy 0 uz 0
              point_for_site Br2 ux 0 uy =vez2; uz 0
              point_for_site Br3 ux =-vez1; uy 0 uz 0
```

```
point_for_site Br4 ux 0 uy =-vez2; uz 0
rotate rotrans  184.60998`_0.40505 qa 1
rotate rotrans  26.81845`_0.33628 qb 1
rotate rotrans  89.47673`_0.56237 qc 1
translate ta rotrans  0.50588`_0.00160 tb 0.00177 tc rotrans  0.73580`_0.00173
prm beq3  1.00000`_0.74665_LIMIT_MIN_1 min 1 max 10
site     C3   x   0.08529`_0.01880   y  0.56093`_0.04924   z
0.36170`_0.03727     occ C   1 beq =beq3;
site     C1        x   0.21761`_0.00934   y  0.45935`_0.02304   z
0.63478`_0.02477     occ C   1 beq =beq3;
site     C2   x   0.11642`_0.00745   y  0.55260`_0.03477   z
0.54100`_0.03693     occ C       1 beq =beq3;
site     N1   x   0.31934`_0.00745   y  0.55229`_0.01503   z
0.63998`_0.01168     occ N       1      beq =beq3;
site     H1        x  0.35135`_0.03571   y      0.58337`_0.09030
z     0.75073`_0.02107    occ H   1     beq =beq3*1.5;
site     H2        x  0.36337`_0.02907   y      0.48663`_0.03806
z     0.60108`_0.10357    occ H   1     beq =beq3*1.5;
site     H3        x  0.30360`_0.01160   y      0.64196`_0.05746
z     0.57123`_0.08657    occ H   1     beq =beq3*1.5;
site     H4        x  0.21640`_0.01365   y      0.43724`_0.03073
z     0.76533`_0.02920    occ H   1     beq =beq3*1.5;
site     H5        x  0.21903`_0.01596   y      0.34699`_0.02117
z     0.56849`_0.03001    occ H   1     beq =beq3*1.5;
site     H6        x  0.07045`_0.01497   y      0.61064`_0.05079
z     0.61441`_0.04719    occ H   1     beq =beq3*1.5;
site     H7        x  0.13125`_0.03107   y      0.50288`_0.06578
z     0.28829`_0.02814    occ H   1     beq =beq3*1.5;
site     H8        x  0.01533`_0.01961   y      0.62539`_0.05703
z     0.29687`_0.04797    occ H   1     beq =beq3*1.5;
normalize_FCs
prm kut1  111.06743`_56.56944_LIMIT_MIN_0 min 0 max 120
rigid
    z_matrix N1
    z_matrix C1 N1 1.453
    z_matrix C2 C1 1.461 N1 113.6
    z_matrix C3 C2 1.31 C1 120 N1 @  76.18973`_14.54713
    z_matrix H1 N1 0.86 C1 109.5 C2 =kut1;
    z_matrix H2 N1 0.86 C1 109.5 C2 =kut1+120;
    z_matrix H3 N1 0.86 C1 109.5 C2 =kut1+240;
    z_matrix H4 C1 1.01 C2 108.5 N1 120
    z_matrix H5 C1 1.01 C2 108.5 N1 -120
    z_matrix H6 C2 1.01 C3 120 C1 -180
    z_matrix H7 C3 1.01 C2 120 C1 0
    z_matrix H8 C3 1.01 C2 120 C1 180
```

```
                    rotate rotrans  103.28588`_7.17613 qa 1
                    rotate rotrans  142.60294`_5.42596 qb 1
                    rotate rotrans  305.49463`_5.11796 qc 1
                    translate ta rotrans  0.31934`_0.00745 tb rotrans  0.55229`_0.01503 tc
rotrans  0.63998`_0.01168


            site          C31    x   0.95242`_0.00753  y  0.55142`_0.02745   z
0.94273`_0.05843              occ C  1 beq =beq3;
            site          C11           x   0.75453`_0.00764  y  0.51496`_0.01471   z
        0.87695`_0.02363            occ C  1 beq =beq3;
            site          C21    x   0.85418`_0.00693  y  0.62260`_0.02043   z
0.91565`_0.03735              occ C         1 beq =beq3;
            site          N11    x   0.65134`_0.00690  y  0.61254`_0.01204   z
0.82934`_0.01409              occ N         1        beq =beq3;
            site          H11           x  0.61239`_0.03020   y      0.58416`_0.07409
       z       0.72115`_0.04433     occ H  1      beq =beq3*1.5;
            site          H21           x  0.61434`_0.03125   y      0.59051`_0.07755
       z       0.90687`_0.06375     occ H  1      beq =beq3*1.5;
            site          H31           x  0.66614`_0.00790   y      0.72079`_0.01260
       z      0.83177`_0.10492      occ H  1      beq =beq3*1.5;
            site          H41           x  0.75437`_0.01181   y      0.43707`_0.01906
       z       0.77032`_0.02998     occ H  1      beq =beq3*1.5;
            site          H51           x  0.75667`_0.01143   y      0.44458`_0.01987
       z      0.98975`_0.02673      occ H  1      beq =beq3*1.5;
            site          H61           x  0.84733`_0.00851   y      0.75191`_0.02001
       z       0.92154`_0.04695     occ H  1      beq =beq3*1.5;
            site          H71           x  0.95927`_0.01008   y      0.42211`_0.02821
       z      0.93684`_0.07177      occ H  1      beq =beq3*1.5;
            site          H81           x  1.02130`_0.00734   y      0.62583`_0.03254
       z      0.96949`_0.07003      occ H  1      beq =beq3*1.5;
            prm kut11  119.99683`_55.04315_LIMIT_MIN_0 min 0 max 120
            rigid
                    z_matrix N11
                    z_matrix C11 N11 1.453
                    z_matrix C21 C11 1.461 N11 113.6
                    z_matrix C31 C21 1.31 C11 120 N11 @  186.70714`_9.78553
                    z_matrix H11 N11 0.86 C11 109.5 C21 =kut11;
                    z_matrix H21 N11 0.86 C11 109.5 C21 =kut11+120;
                    z_matrix H31 N11 0.86 C11 109.5 C21 =kut11+240;
                    z_matrix H41 C11 1.01 C21 108.5 N11 120
                    z_matrix H51 C11 1.01 C21 108.5 N11 -120
                    z_matrix H61 C21 1.01 C31 120 C11 -180
                    z_matrix H71 C31 1.01 C21 120 C11 0
                    z_matrix H81 C31 1.01 C21 120 C11 180
                    rotate rotrans  269.76423`_5.62598 qa 1
```

```
rotate rotrans  161.78410`_7.19099 qb 1

rotate rotrans  238.54743`_3.01179 qc 1

translate ta rotrans  0.65134`_0.00690 tb rotrans  0.61254`_0.01204 tc rotrans  0.82934`_0.01409
```

$aacn_2CuBr_4$

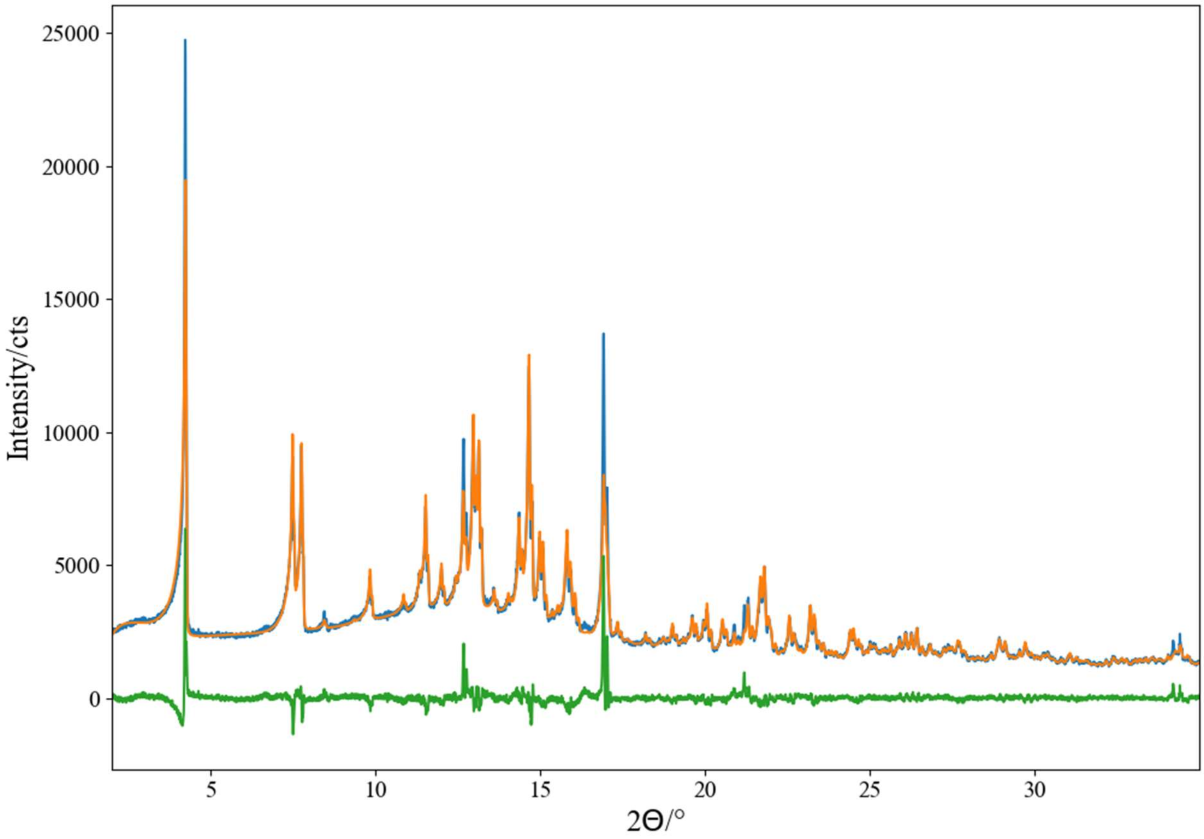

Figure S15. PXRD data for **$aacn_2CuBr_4$** (blue), calculated pattern (orange) and difference between observed and calculated pattern (green).

```
xdd aacn2cubr4.xy

macro rotrans { @ }

do_errors

randomize_on_errors

iters 10000

       r_wp  6.16747841 r_exp  1.90352012 r_p  3.93435105 r_wp_dash  18.4734158 r_p_dash  15.3143446 r_exp_dash  5.70160386 weighted_Durbin_Watson  0.253414402 gof  3.24003846

       bkg @  1976.22544`_5.88978298 -881.726793`_7.69887091 -194.50276`_5.69114969 319.938392`_5.28550509  45.5993554`_5.19766738 -262.81402`_5.13724438  123.065113`_5.03579521 107.981978`_5.07216927 -142.686061`_4.97980239  25.2843412`_4.70161972  4.44953414`_4.73990428 -13.1589755`_4.66754017 -57.1616906`_5.2014443  69.553838`_4.88744362 -39.0164792`_4.77564132 -9.48991725`_4.66450975 -2.79184833`_4.41351636  14.1956173`_3.92547382 6.27250605`_3.78335322

   lam ymin_on_ymax 0.0001

   Lam_recs
```

```
{
   0.6533  0.709300  0.2695
   0.3467  0.713574  0.2795
}
Radius(240)
     Zero_Error(@, 0.03275`_0.00042)
     LP_Factor(0)
     Full_Axial_Model(12, 15, 12, @  3.34752`_0.80802, @  10.28958`_6.19940)
     x_calculation_step 0.01
     xdd_out "aacn2cubr4.txt" load out_record out_fmt out_eqn
{
      " %11.6f  " = X;
     " %11.6f  " = Yobs;
     " %11.6f  " = Ycalc;
     " %11.6f\n" = Yobs-Ycalc;
}
     str
             CS_G(@, 174.23833`_7.84654)
             Strain_L(@, 0.12971`_0.00907)
             a  @  10.076857`_0.000497
             b  @  8.290167`_0.000385
             c  @  7.544772`_0.000345
             be @  106.67643`_0.00368
             space_group "P21/c"
             scale @  5.50323936e-005`_4.736e-007
             prm beq1  2.28346`_0.09832 min 1 max 10
             site Cu1    x  0.00000`_0.00000 y  0.00000`_0.00000 z  0.50000`_0.00000 occ Cu
1 beq =beq1;
             site Br1    x -0.01336`_0.00063 y -0.18045`_0.00060 z  0.23163`_0.00072 occ Br
1 beq =beq1;
             site Br2    x -0.25077`_0.00049 y  0.02182`_0.00161 z  0.40104`_0.00115 occ Br
1 beq =beq1;
             prm veza1  2.48982`_0.00537 min 2.2 max 2.6
             prm veza2  2.42756`_0.00459 min 2.2 max 2.6
             prm kut  89.20186`_0.18831 min 70 max 110
             rigid
             point_for_site Cu1 ux 0 uy 0 uz 0
             point_for_site Br1 ux =veza1; uy 0 uz 0
             point_for_site Br2 ux =veza2*Cos(kut*3.141592/180); uy =
veza2*Sin(kut*3.141592/180); uz 0
                     rotate rotrans  179.78385`_0.22782 min 0 max 359.99 qa 1
```

```
rotate rotrans  36.23442`_0.10876 min 0 max 359.99 qb 1

rotate rotrans  265.07330`_0.23097 min 0 max 359.99 qc 1

translate ta 0 tb 0 tc =1/2;

prm beq2  5.77188`_0.58615_LIMIT_MAX_10 min 1 max 10

site           N1      x   0.51805`_0.01705   y    0.79144`_0.02052  z
0.97352`_0.00641       occ N   1       beq  =beq2;

site           C1             x   0.59124`_0.01550   y    0.85612`_0.01898  z
0.90569`_0.00460      occ C   1       beq  =beq2;

site           C2      x   0.68389`_0.01373   y    0.93738`_0.01734  z
0.81782`_0.00333       occ C   1       beq      =beq2;

site           N2      x   0.75812`_0.01529   y    1.06854`_0.01857  z
0.93708`_0.00472       occ N   1       beq      =beq2;

site           H1      x   0.79680`_0.01651   y    1.03344`_0.01978  z
1.03404`_0.00403       occ H   1       beq      =beq2*1.2;

site           H2      x   0.70315`_0.01607   y    1.12909`_0.01908  z
0.95567`_0.00724       occ H   1       beq      =beq2*1.2;

site           H3             x      0.79806`_0.01449      y
1.10925`_0.01786 z    0.89145`_0.00503             occ H         1             beq
=beq2*1.2;

site           H4             x      0.62658`_0.01251      y
0.97718`_0.01625 z    0.69732`_0.00448             occ H         1             beq
=beq2*1.2;

site           H5             x      0.73848`_0.01297      y
0.85894`_0.01681 z    0.78726`_0.00439             occ H         1             beq
=beq2*1.2;

rigid

point_for_site  N1    ux 1.2635 uy  2.9427 uz  3.8405

point_for_site  C1    ux 1.2615 uy  2.9524 uz  2.6960

point_for_site  C2    ux 1.2584 uy  2.9505 uz  1.2399

point_for_site  N2    ux 1.2574 uy  4.3318 uz  0.7329

point_for_site  H1    ux 1.8674 uy  4.7142 uz  1.0325

point_for_site  H2    ux 0.6230 uy  4.7045 uz  1.0187

point_for_site  H3    ux 1.2118 uy  4.3173 uz  0.0461

point_for_site  H4    ux 0.4619 uy  2.4490 uz  0.9541

point_for_site  H5    ux 1.9628 uy  2.4200 uz  0.9680

rotate rotrans  48.86664`_0.78290 min 0 max 359.99 qa 1

rotate rotrans  329.15650`_1.93310 min 0 max 359.99 qb 1

rotate rotrans  318.93791`_0.74839 min 0 max 359.99 qc 1

translate ta rotrans  0.57814`_0.01026 min 0 max 1 tb rotrans
0.88356`_0.01450 min 0 max 1 tc rotrans  0.34607`_0.00312 min 0 max 1
```

cpa_2CuBr_4

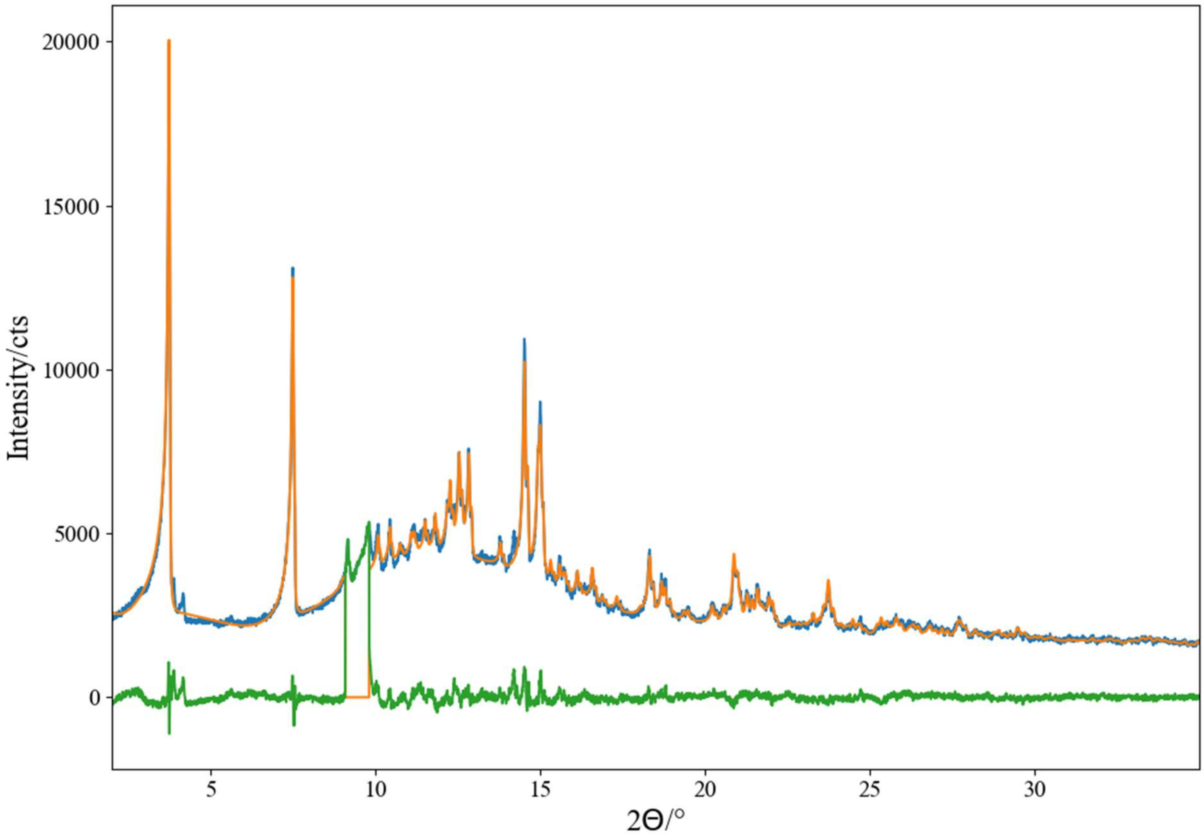

Figure S16. PXRD data for **cpa_2CuBr_4** (blue), calculated pattern (orange) and difference between observed and calculated pattern (green). The region between 9.1 and 9.8 °2θ is excluded from fit.

```
xdd cpa2cubr4_2.xy

iters 50000

macro rotrans {@}


'Auto_T(50)

do_errors

r_wp  4.15452324 r_exp  1.82567102 r_p  3.14009576 r_wp_dash  16.6979934 r_p_dash  18.1564671
r_exp_dash  7.33779568 weighted_Durbin_Watson  0.446936477 gof  2.27561438


       LP_Factor(0)

       Zero_Error(, 0.02681)

       Full_Axial_Model(12, 15, 12,   12.54022_1.17720,    2.16332_0.22014)

       Radius(240)


    lam ymin_on_ymax 0.0001

    Lam_recs

    {
```

```
      0.6533  0.709300  0.2695
      0.3467  0.713574  0.2795
   }

       xdd_out "cpa2cubr4_2.txt" load out_record out_fmt out_eqn
   {
        " %11.6f  " = X;
      " %11.6f  " = Yobs;
      " %11.6f  " = Ycalc;
      " %11.6f\n" = Yobs-Ycalc;
   }
       bkg  @   2335.77842`_2.95440024 -630.883542`_4.49910149 -512.663914`_3.85848496
454.84835`_3.73423097  67.8298926`_3.46393095 -367.594261`_3.47563636  200.927858`_3.51012278
119.071507`_3.49905454 -251.740246`_3.53232696  102.812889`_3.37935809  83.5251763`_3.18372989
-137.342734`_2.90371  56.8793076`_2.88341949
       x_calculation_step 0.01

       exclude 9.1 9.8
       str
       'hkl_Is

               CS_L(@, 206.96960`_6.44566)
               Strain_G(@, 0.26589`_0.00780)
               a @   7.806465`_0.000453
               b @   8.085963`_0.000517
               c @   21.862873`_0.000901
               space_group "Bmab"
               scale @  8.7384532e-006`_5.242e-008
               prm beq1  2.31929`_0.10754 min 1 max 10
               site Cu1 x  0.00000`_0.00000 y  0.00000`_0.00000 z  0.00000`_0.00000 occ Cu 1
beq =beq1;
               site Br1 x -0.02072`_0.00096 y -0.04576`_0.00052 z  0.10994`_0.00020 occ Br 0.5
beq =beq1;
               site Br2 x  0.22349`_0.00619 y  0.21199`_0.00592 z  0.01744`_0.00018 occ Br 0.5
beq =beq1;
               prm vez1  2.43730`_0.00441 min 2 max 3
               prm vez2  2.47539`_0.00599 min 2 max 3
               'prm vez3  2.52641 min 2 max 3
               'prm vez4  2.72481 min 2 max 3
               normalize_FCs

               rigid
```

```
point_for_site Cu1 ux 0 uy 0 uz 0
point_for_site Br1 ux =vez1; uy 0 uz 0
point_for_site Br2 ux 0 uy =vez2; uz 0
rotate rotrans  68.37735`_1.08848 qa 1
rotate rotrans  279.53698`_0.06894 qb 1
rotate rotrans  246.39151`_1.14532 qc 1
translate ta 0 tb 0 tc 0

prm beq2  2.68750` min 1 max 10
site N1  x  0.49293`_0.00690 y  0.00110`_0.00328 z  0.10926`_0.00061 occ N 0.5 beq =beq1;
site C1  x  0.42362`_0.00843 y -0.02192`_0.00535 z  0.17268`_0.00090 occ C 0.5 beq =beq1;
site C2  x  0.40452`_0.01080 y  0.14422`_0.00684 z  0.20243`_0.00146 occ C 0.5 beq =beq1;
site C3  x  0.55153`_0.01063 y  0.03227`_0.00715 z  0.21979`_0.00086 occ C 0.5 beq =beq1;

rigid
z_matrix N1
z_matrix C1 N1 1.5
z_matrix C2 C1 1.5 N1 109
z_matrix C3 C1 1.5 N1 111 C2 -64.75
rotate rotrans  159.00694`_1.52026 qa 1
rotate rotrans  188.06810`_1.15152 qb 1
rotate rotrans  268.89474`_2.09522 qc 1
translate ta rotrans  0.49293`_0.00690 tb rotrans  0.00110`_0.00328 tc rotrans 0.10926`_0.00061
```

$cpma_2CuBr_4$

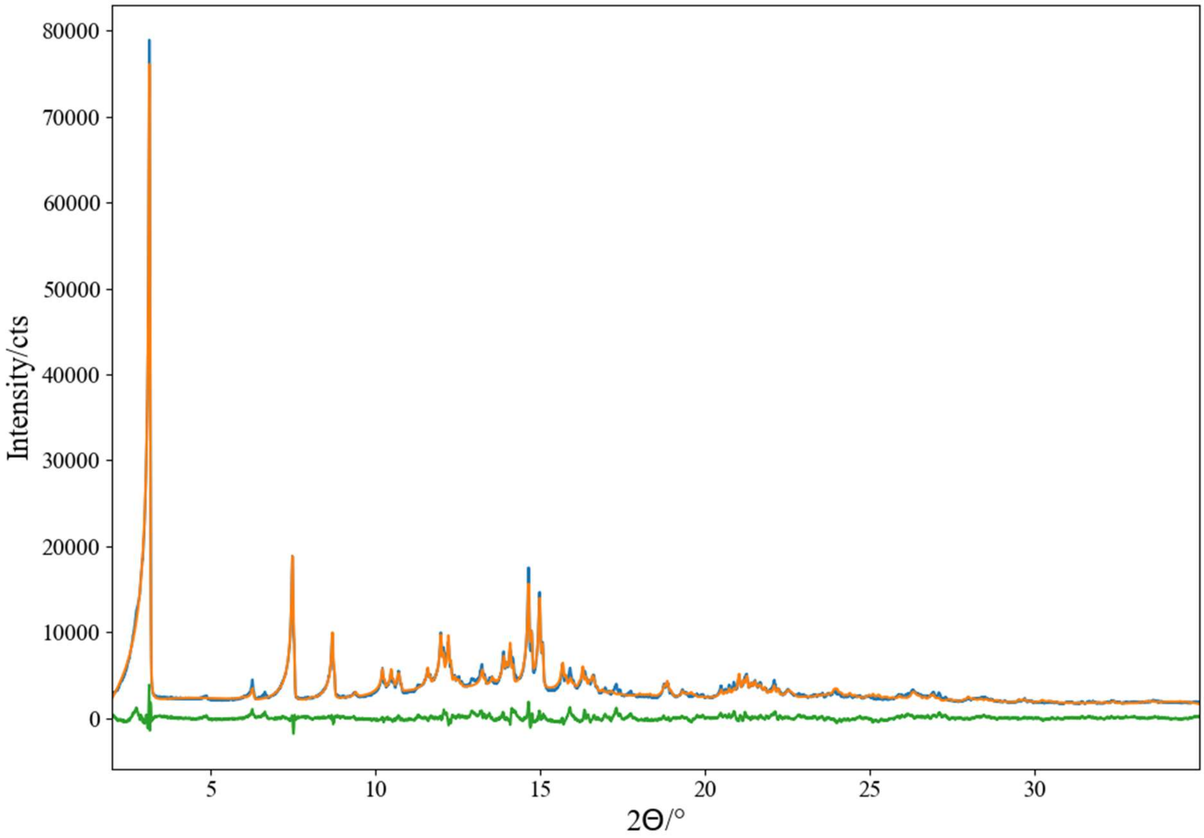

Figure S17. PXRD data for **$cpma_2CuBr_4$** (blue), calculated pattern (orange) and difference between observed and calculated pattern (green). The region between 9.1 and 9.8 °2θ is excluded from fit.

```
xdd cpma2cubr4.xy

iters 20000

macro rotrans {@}

'Auto_T(100)

do_errors

r_wp  6.52331649 r_exp  1.68338677 r_p  4.99345988 r_wp_dash  14.1964309 r_p_dash  14.6879366
r_exp_dash  3.66348684 weighted_Durbin_Watson  0.17261235 gof  3.87511449


       LP_Factor(0)

       Zero_Error(@, 0.02220`_0.00034)

       Full_Axial_Model(12, 15, 12,  @  8.58440`_0.49897,  @  3.87056`_0.12299)

       Radius(240)


   lam ymin_on_ymax 0.0001

   Lam_recs

   {

      0.6533  0.709300  0.2695
```

```
      0.3467  0.713574  0.2795
   }

        xdd_out "cpma2cubr4.txt" load out_record out_fmt out_eqn
   {
         " %11.6f  " = X;
       " %11.6f  " = Yobs;
       " %11.6f  " = Ycalc;
       " %11.6f\n" = Yobs-Ycalc;
   }
        bkg  @   2019.71473`_9.82206666  61.3242429`_15.9030023 -837.07247`_12.2894465
547.425597`_10.6945783 -117.311334`_9.86035952  77.8691445`_9.97456126 -174.129282`_8.64008443
314.000814`_8.22058772 -327.476179`_7.35794565  50.2648207`_7.07821717  80.9584211`_6.50657278
-55.7167419`_6.20074236 -51.7505296`_6.38691517
        x_calculation_step 0.01
        str
                CS_L(@, 555.93461`_74.51451)
                CS_G(@, 167.10131`_6.32446)
                Strain_G(@, 0.06936`_0.05066_LIMIT_MIN_0.0001)
                Strain_L(@, 0.09507`_0.02064)
                a  @  7.775261`_0.000371
                b  @  7.977310`_0.000449
                c  @  26.002141`_0.002189
                space_group "B2cb"
                scale @  1.37598442e-005`_2.417e-007
                'lebail 1
                prm beq1  5.77675`_0.18459 min 1 max 10
                site Cu1 x  0.87334`_0.00000 y  0.00000`_0.00000 z  0.00000`_0.00000 occ Cu 1
beq =beq1;
                site Br1 x  0.84120`_0.00172 y  0.04392`_0.00140 z  0.09080`_0.00033 occ Br 1
beq =beq1;
                site Br2 x  1.08860`_0.01215 y -0.21927`_0.01148 z  0.01679`_0.00033 occ Br 1
beq =beq1;
                prm vez1  2.40000`_0.00852_LIMIT_MIN_2.4 min 2.40 max 2.46
                prm vez2  2.46000`_0.01438_LIMIT_MAX_2.46 min 2.40 max 2.46
                'prm vez3  2.35694 min 2.2 max 2.5
                'prm vez4  2.20024 min 2.2 max 2.5
                normalize_FCs
                rigid
                point_for_site Cu1 ux 0 uy 0 uz 0
                point_for_site Br1 ux =vez1; uy 0 uz 0
                point_for_site Br2 ux 0 uy =vez2; uz 0
```

```
'point_for_site Br3 ux =-vez3; uy 0 uz 0
		'point_for_site Br4 ux 0 uy =-vez4; uz 0
		rotate rotrans  278.10852`_2.27088 qa 1
		rotate rotrans  259.67027`_0.18788 qb 1
		rotate rotrans  305.49552`_2.06415 qc 1
		translate ta 0.87334 tb 0 tc 0
		prm beq2  7.13855`_1.14110_LIMIT_MAX_10 min 1 max 10
		site N1  x  0.30776`_0.00764 y  0.48661`_0.00656 z  0.41382`_0.00120 occ N 1 
beq =beq2;
		site C1  x  0.27610`_0.01147 y  0.40594`_0.00867 z  0.36432`_0.00141 occ C 1 
beq =beq2;
		site C2  x  0.35887`_0.01255 y  0.50017`_0.01119 z  0.32200`_0.00128 occ C 1 
beq =beq2;
		site C3  x  0.54738`_0.01257 y  0.53061`_0.02275 z  0.32635`_0.00364 occ C 1 
beq =beq2;
		site C4  x  0.47702`_0.02255 y  0.40474`_0.01664 z  0.28767`_0.00396 occ C 1 
beq =beq2;
		site H1 x  0.31507`_0.11567 y  0.59328`_0.01169 z  0.40957`_0.00534 occ H 1 beq 
=beq2*1.5;
		site H2 x  0.22446`_0.05712 y  0.46453`_0.09338 z  0.43450`_0.01112 occ H 1 beq 
=beq2*1.5;
		site H3 x  0.40245`_0.05920 y  0.44961`_0.08384 z  0.42660`_0.01622 occ H 1 beq 
=beq2*1.5;
		site H4 x  0.32474`_0.01717 y  0.28858`_0.00811 z  0.36519`_0.00224 occ H 1 beq 
=beq2*1.5;
		site H5 x  0.14802`_0.01208 y  0.40159`_0.01402 z  0.35797`_0.00199 occ H 1 beq 
=beq2*1.5;
		site H6 x  0.28962`_0.01509 y  0.59244`_0.01504 z  0.30530`_0.00288 occ H 1 beq 
=beq2*1.5;
		site H7 x  0.59227`_0.01803 y  0.64041`_0.02564 z  0.31243`_0.00349 occ H 1 beq 
=beq2*1.5;
		site H8 x  0.60687`_0.01117 y  0.48523`_0.03118 z  0.35795`_0.00524 occ H 1 beq 
=beq2*1.5;
		site H9 x  0.47984`_0.02715 y  0.43856`_0.01892 z  0.25025`_0.00354 occ H 1 beq 
=beq2*1.5;
		site H10 x  0.49224`_0.03162 y  0.28141`_0.01740 z  0.29519`_0.00609 occ H 1 
beq =beq2*1.5;
		prm kut  55.80683`_6.00924 min 0 max 360
		prm kut2  31.96447`_64.23409_LIMIT_MIN_0 min 0 max 120
		rigid
		z_matrix N1
		z_matrix C1 N1 1.46
		z_matrix C2 C1 1.48 N1   111
		z_matrix C3 C2 1.49 C1 117 N1 =kut;
		z_matrix C4 C2 1.49 C1 117 N1 =kut+70;
		z_matrix H1 N1 0.86 C1 109.5 C2 =kut2;
```

```
z_matrix H2 N1 0.86 C1 109.5 C2 =kut2+120;
z_matrix H3 N1 0.86 C1 109.5 C2 =kut2+240;
z_matrix H4 C1 1.01 N1 109    C2 120
z_matrix H5 C1 1.01 N1 109    C2 -120
z_matrix H6 C2 1.01 C3 116 C4 -107.25
z_matrix H7 C3 1.01 C2 117 H6 0
z_matrix H8 C3 1.01 C2 117 H6 145
z_matrix H9 C4 1.01 C2 117 H6 0
z_matrix H10 C4 1.01 C2 117 H6 -145
rotate rotrans  172.66073`_2.31806 qa 1
rotate rotrans  27.26163`_1.54523 qb 1
rotate rotrans  53.35634`_3.54816 qc 1
translate ta rotrans  0.30776`_0.00764 tb rotrans  0.48661`_0.00656 tc rotrans 0.41382`_0.00120
```

Refinements with crystal structure from SCXRD data

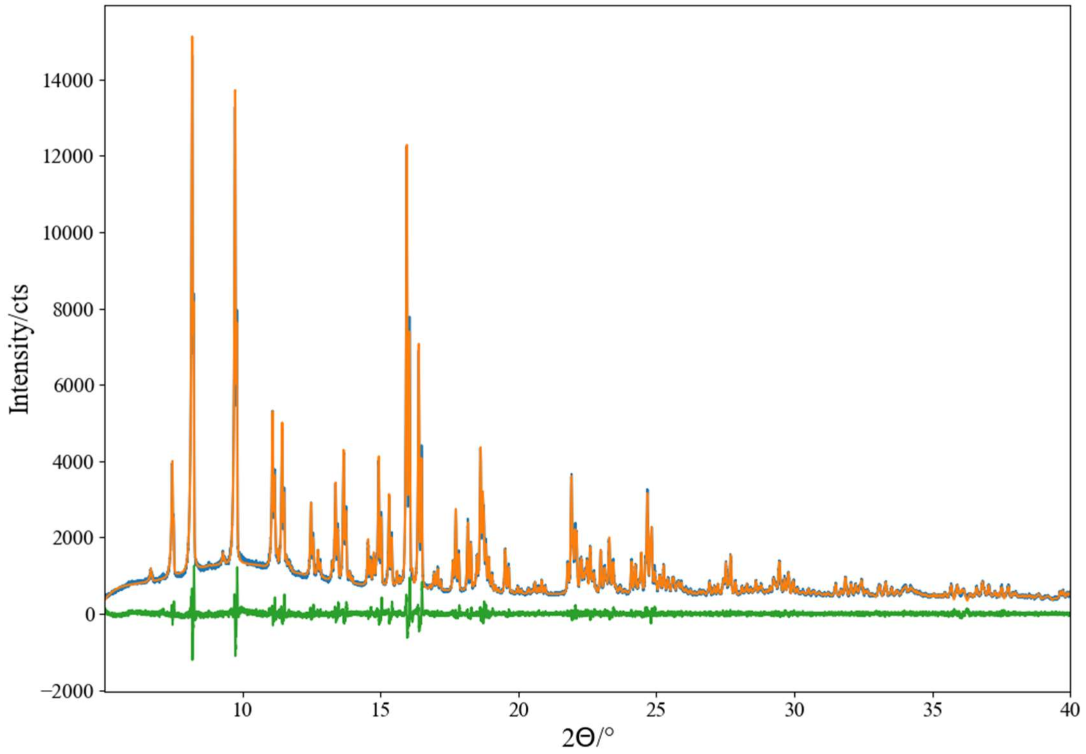

Figure S18. PXRD data (Mo Kα) for **aacn$_2$CuCl$_4$** (blue), calculated pattern (orange) and difference between observed and calculated pattern (green). The fit was done on crystal structure obtained from the SCXRD experiments. R_{wp} = 5.06%.

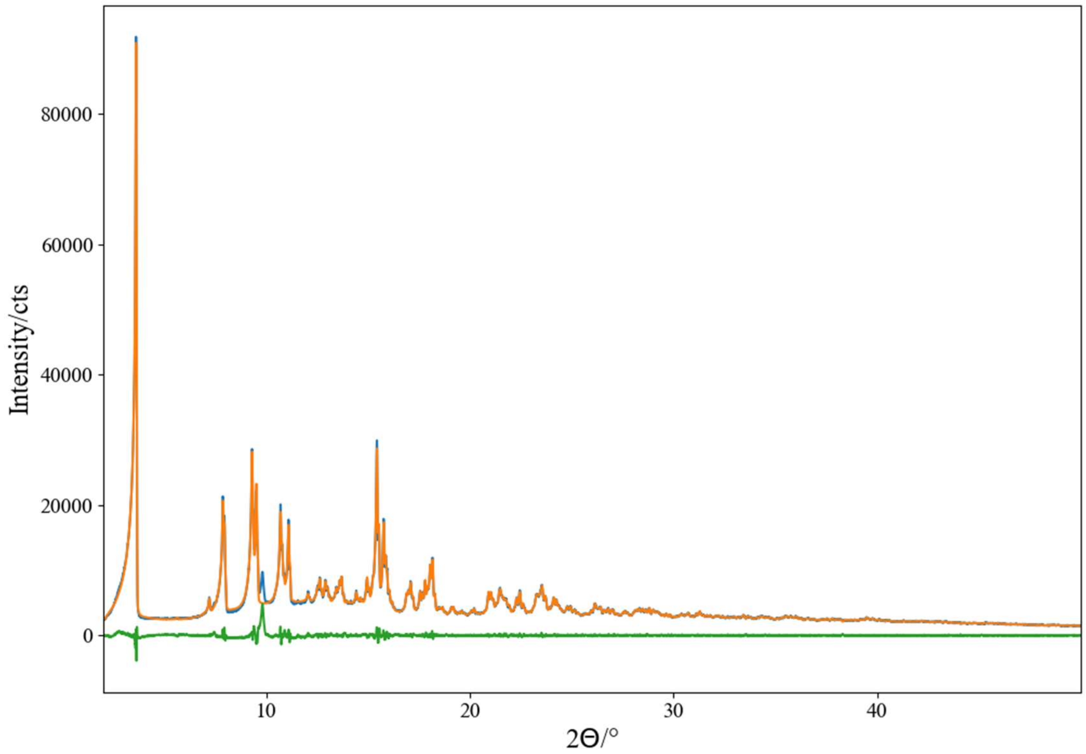

Figure S19. PXRD data (Mo Kα) for **cpa_2CuCl_4** (blue), calculated pattern (orange) and difference between observed and calculated pattern (green). The fit was done on crystal structure obtained from the SCXRD experiments. R_{wp} = 4.84%.

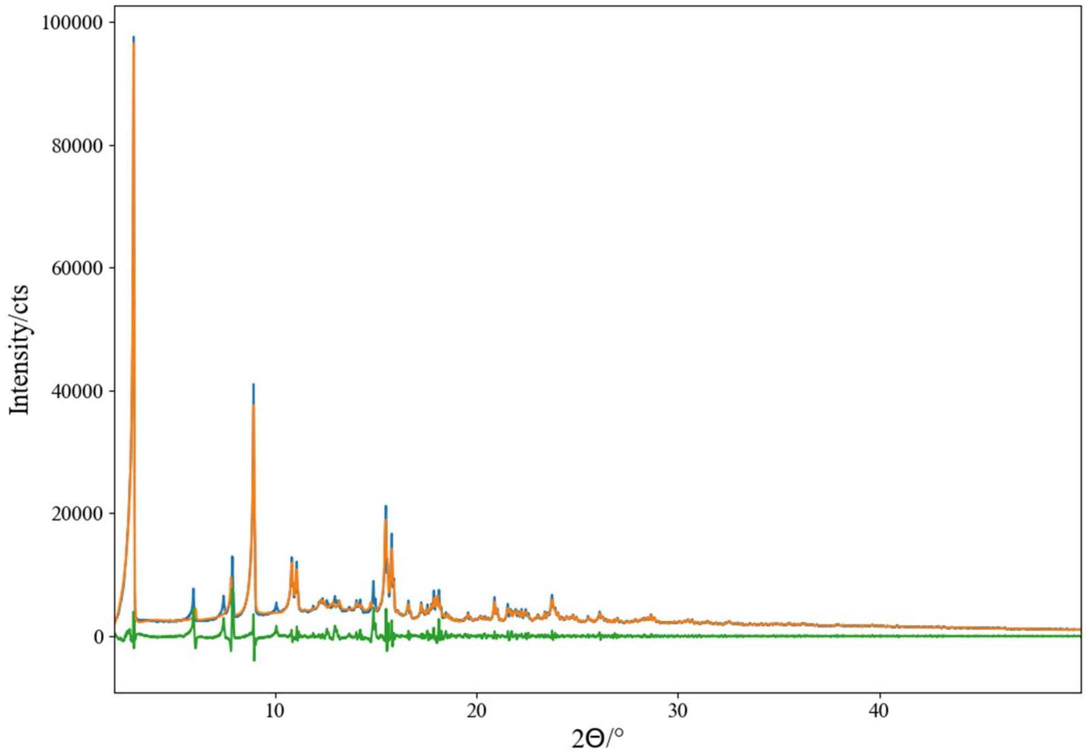

Figure S20. PXRD data (Mo Kα) for **$cpma_2CuCl_4$** (blue), calculated pattern (orange) and difference between observed and calculated pattern (green). The fit was done on crystal structure obtained from the SCXRD experiments. R_{wp} = 9.57%.

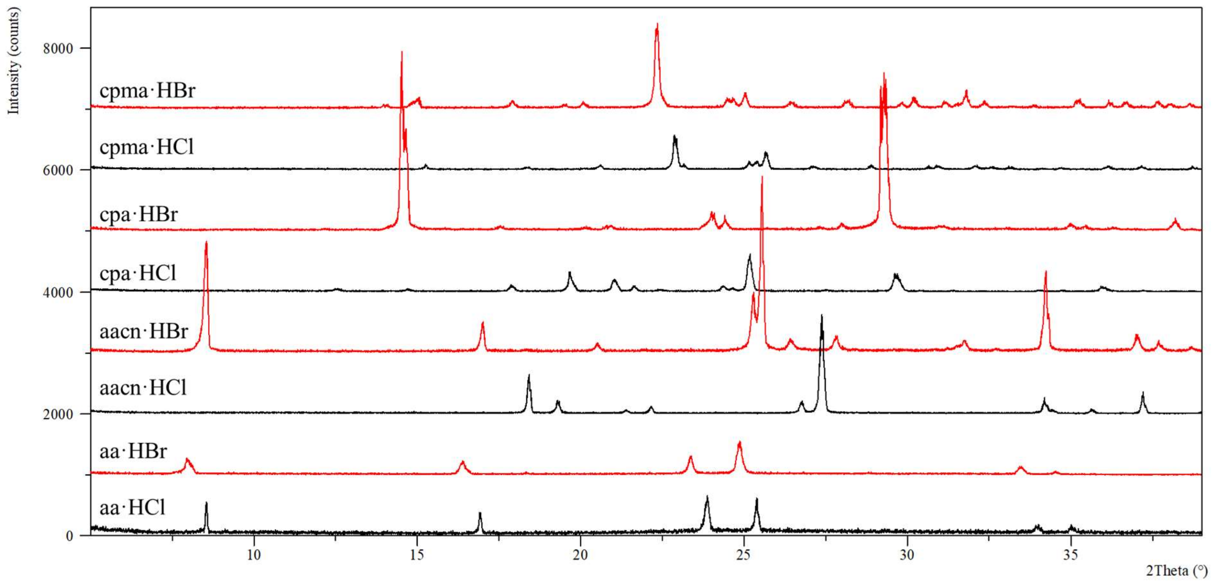

Figure S21. PXRD data (Cu Kα) of alkylammonium halogenides. Diffraction patterns of chloride salts are shown as black lines, while those of bromide salts are shown as red lines.

Thermal analysis

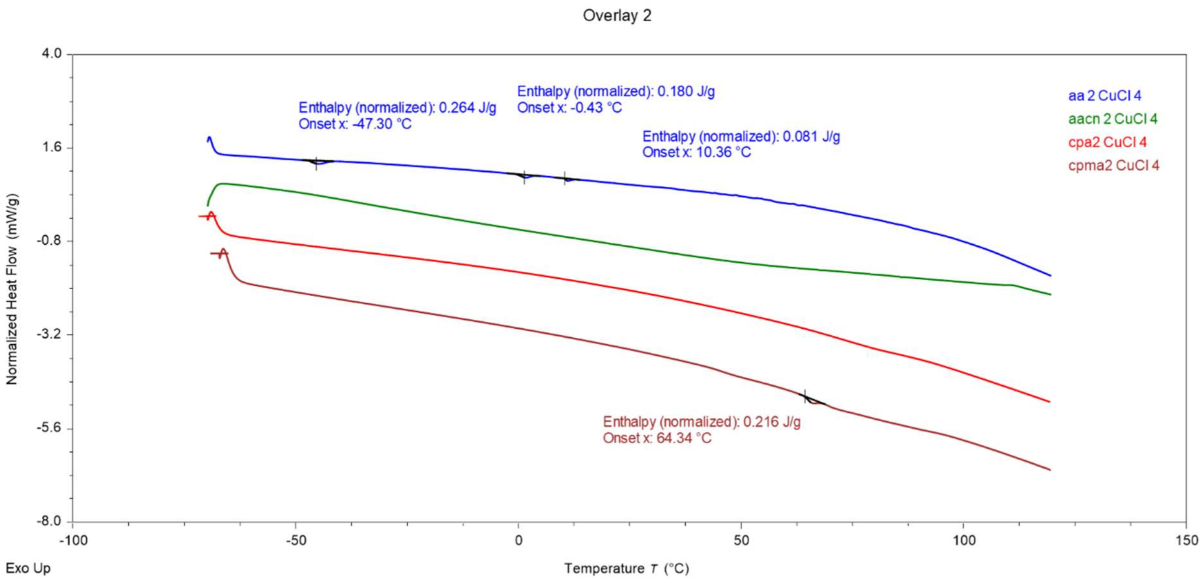

Figure S22. DSC thermograms of **aa$_2$CuCl$_4$** (blue), **aacn$_2$CuCl$_4$** (green), **cpa$_2$CuCl$_4$** (red) and **cpma$_2$CuCl$_4$** (dark red). While **aacn$_2$CuCl$_4$** and **cpa$_2$CuCl$_4$** show no thermal events in the probed temperature range, **aa$_2$CuCl$_4$** and **cpma$_2$CuCl$_4$** show a few weak endothermic events indicating possible structural phase transitions, albeit the structural changes should be minimal considering the enthalpies of thermal events are minute (less than 1 kJ mol^{-1}).

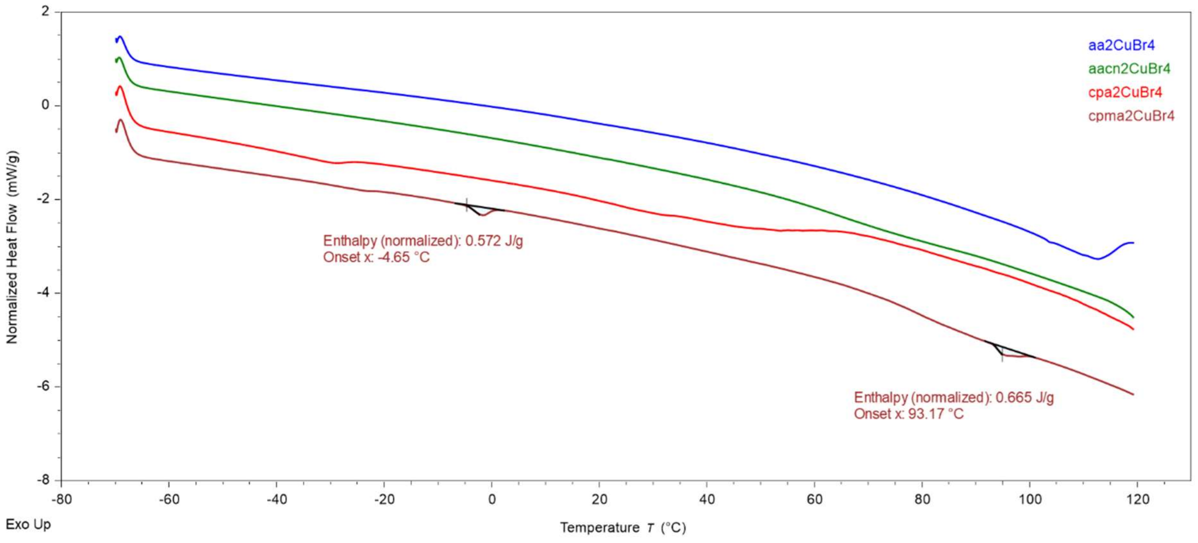

Figure S23. DSC thermograms of **aa$_2$CuBr$_4$** (blue), **aacn$_2$CuBr$_4$** (green), **cpa$_2$CuBr$_4$** (red) and **cpma$_2$CuBr$_4$** (dark red). As with chlorocuprates(II), no proper thermal events are observed except in **cpma$_2$CuBr$_4$** with two weak endothermal events with enthalpies less than 1 kJ mol^{-1}.
